# SOX9 Protein in Pancreatic Cancer Regulates Multiple Cellular Networks in a Cell-Specific Manner

**DOI:** 10.3390/biomedicines10071466

**Published:** 2022-06-21

**Authors:** Eugene Kopantzev, Liya Kondratyeva, Marina Kopantseva, Kirill Kashkin, Dmitry Gnatenko, Elizaveta Grigorieva, Irina Alekseenko, Dina Safina, Igor Chernov

**Affiliations:** 1 Shemyakin-Ovchinnikov Institute of Bioorganic Chemistry, Russian Academy of Sciences, Ulitsa Miklukho-Maklaya, 117997 Moscow, Russia; ekopantzev@mail.ru (M.K.); kachkine@yandex.ru (K.K.); gnatenkodmitrij@gmail.com (D.G.); grigorieva.es@phystech.edu (E.G.); irina.alekseenko@mail.ru (I.A.); igor.palich@gmail.com (I.C.); 2 Institute of Molecular Genetics of National Research Centre “Kurchatov Institute”, Ploshchad’ Akademika Kurchatova, 123182 Moscow, Russia; nauruz@mail.ru

**Keywords:** pancreatic ductal adenocarcinoma, transcription factor, SOX9, differentiation markers, cell proliferation, apoptosis

## Abstract

SOX9 is upregulated in the majority of pancreatic ductal adenocarcinoma cases. It is hypothesized that the increased expression of SOX9 is necessary for the formation and maintenance of tumor phenotypes in pancreatic cancer cells. In our research, we studied six pancreatic cancer cell lines, which displayed varying levels of differentiation and a range of oncogenic mutations. We chose the method of downregulation of SOX9 expression via siRNA transfection as the main method for investigating the functional role of the SOX9 factor in pancreatic cancer cells. We discovered that the downregulation of SOX9 expression in the cell lines leads to cell-line-specific changes in the expression levels of epithelial and mesenchymal protein markers. Additionally, the downregulation of SOX9 expression had a specific effect on the expression of pancreatic developmental master genes. SOX9 downregulation had the greatest effect on the expression levels of the protein regulators of cell proliferation. In three of the four cell lines studied, the transfection of siSOX9 led to a significant decrease in proliferative activity and to the activation of proapoptotic caspases in transfected cells. The acquired results demonstrate that the SOX9 protein exerts its multiple functions as a pleiotropic regulator of differentiation and a potential promoter of tumor growth in a cell-specific manner in pancreatic cancer cells.

## 1. Introduction

Pancreatic cancer is the fourth leading cause of cancer-related mortality in industrial Western countries [1]. Pancreatic ductal adenocarcinoma (PDAC) is the most common type of exocrine pancreatic cancer, accounting for 90% of pancreatic cancer diagnoses [2]. The majority of PDAC patients are diagnosed with terminal-stage disease and are insensitive to chemotherapy. Therapeutic options for patients with locally advanced or advanced PDAC are extremely limited, and the 5-year median survival rate is below 5%. The current standard-of-care chemotherapy for unresectable or metastatic PDAC, such as gemcitabine monotherapy, gemcitabine combined with nab-paclitaxel, or FOLFIRINOX combination chemotherapy, extends patient survival by only a few months [3]. Consequently, understanding the cellular biology of PDAC and its metastasis may provide insight for the development of novel molecular targets and new effective therapeutic strategies. PDAC is widely regarded as a genetic disease associated with activating mutations of the KRAS oncogene in early precursor lesions (pancreatic intraepithelial neoplasia (PanIN-1)) and inactivating mutations of the tumor suppressor genes CDKN2A, TP53, and SMAD4 accumulating in more late and advanced premalignant lesions (PanIN-2 and PanIN-3) during PDAC initiation and maintenance [4,5]. Driver mutations of the KRAS, CDKN2A, TP53, and SMAD4 genes in PDAC precursor cells induce multiple changes in the expression of genes that maintain uncontrolled proliferation and survival of cancer cells. This consequently contributes to the irreversible nature of pancreatic tumor growth [6,7]. SOX9 is one of the genes whose deregulated expression plays an important role in the initiation and development of PDAC [8,9].

SOX9 (sex-determining region Y-box protein 9) is a member of the SOX transcription factor family. It is an evolutionarily conserved regulator of many developmental processes, including chondrogenesis, bone formation, and testis development [10]. Deregulation of the SOX9 expression has been implicated in carcinogenesis in different organs and tissues. It has been shown that SOX9 is overexpressed in cancers of the skin, prostate, lung, and breast and contributes to tumor growth and invasion [11,12,13,14]. Increased SOX9 expression has been associated with clinical stage and poor prognosis in these cancers. However, SOX9 is a tumor suppressor in cervical cancer, endometrial carcinoma, melanomas, and some intestinal tumors [15,16,17,18]. During early pancreatic development, SOX9 plays a pivotal role in controlling pancreatic epithelial progenitor cell proliferation, while in mature pancreas, SOX9 maintains ductal integrity [19]. Recent research indicates that SOX9 is essential during acinar to ductal metaplasia initiation and is indispensable for the formation of PanIN lesions induced by oncogenic KRAS expression [8]. Furthermore, SOX9 is upregulated in the majority of PDAC cases associated with poor patient survival. It is hypothesized that the increased expression of SOX9 is necessary for the formation and maintenance of the tumor phenotype in pancreatic cancer cells. The biological significance of deregulated SOX9 expression in the pathologic transformation of cellular processes and signaling pathways in pancreatic cancer cells has not been extensively studied yet. The role of this transcription factor in pancreatic carcinogenesis requires further investigations.

Pancreatic tumors are frequently characterized by a high level of genetic and phenotypic heterogeneity between cancer cells [20]. Therefore, taking into account the significant intratumoral and intertumoral heterogeneity of PDAC, we conducted a relative assessment of changes that occur after the downregulation of SOX9 expression via siRNA in different pancreatic cancer cell lines. The investigated cell lines were characterized by varying genotypes and differentiation levels. The acquired results demonstrate that the SOX9 protein exerts its multiple functions as a regulator of differentiation and a potential promoter of tumor growth in a cell-specific manner in pancreatic cancer cells. This important fact should be taken into account during the development of potential strategies for therapeutic targeting of SOX9 and other similar transcription factors.

## 2. Materials and Methods

### 2.1. Materials and Cell Cultures

Unless otherwise specified, the chemicals were obtained from Sigma-Aldrich (Sigma-Aldrich, St. Louis, MO, USA). Sera and cell culture media were obtained from Invitrogen (Invitrogen Corporation, Carlsbad, CA, USA). The list of primary and secondary antibodies used in experiments is presented in Supplementary Material Table S1. AsPC-1, BxPC-3, Capan-2, Mia PaCa-2, and Panc1 human pancreatic cancer cell lines were obtained from the American Type Cell Collection (ATCC, Manassas, VA, USA), and Colo357 [21] was obtained from Dr. Klaus Felix (University Hospital Heidelberg, Heidelberg, Germany). AsPC-1, Capan-2, Mia PaCa-2, and Panc1 cells were maintained in Dulbecco’s modified Eagle medium/Ham’s F12 (DMEM/F12, 1:1) containing 10% fetal calf serum, 2 mM L-glutamine, 100 U/mL penicillin, and 100 µg/mL streptomycin. BxPC-3 and Colo357 cells were maintained in RPMI 1640 medium containing 10% fetal calf serum, 2 mM L-glutamine, 55 nM 2-mercaptoethanol, 100 U/mL penicillin, and 100 µg/mL streptomycin. All cells were cultured at 37 °C in a humidified atmosphere of 95% air and 5% CO_2_.

### 2.2. Knockdown of SOX9 via RNA Interference

Negative control #2 siRNA (Cat #4390846, Lot #AS02F4T6) was purchased from Ambion, Inc. (Austin, TX, USA). The siRNAs targeting human SOX9 were synthesized by Syntol (Syntol JSC, Moscow, Russia). The sequences of sense strands of SOX9 siRNAs are presented in Supplementary Material Table S2. Three different SOX9 siRNA duplexes were tested individually and in a combination. siRNA transfections were carried out using the Lipofectamine RNAiMAX reagent (Invitrogen), following the manufacturers protocol. Pancreatic cancer cells seeded in 6-well plates were transfected with 10 nM siSOX9 pooled duplexes and control 10 nM siNeg. To achieve the best depletion of the SOX9 protein, transfection was conducted twice: 24 h after seeding the cells and 48 h after the first transfection. Seventy-two hours after the first transfection, cells were harvested for further analysis.

### 2.3. Plasmid DNA Transfection

pCMV6-SOX9 human tagged ORF clone (RC208994) and control pCMV6-Entry plasmid vector (PS100001) were obtained from OriGene Technologies (Rockville, MD, USA). Plasmid DNA transfections were carried out using the Lipofectamine 3000 reagent (Invitrogen), following the manufacturers protocol. After 72 h of incubation, cells were harvested for further analysis.

### 2.4. Western Blot Analysis

Cell lysates were prepared from siSOX9 transfected experimental cells and siNeg transfected control cells, according to the protocol described earlier [22]. Cell lysates were boiled in SDS sample buffer consisting of 1% SDS, 2% 2-mercaptoethanol, and 62 mM Tris-HCl, pH 6.8. Equal amounts of denatured protein lysates (20 µg of total protein) were subjected to SDS electrophoresis in 10%–15% polyacrylamide gels and then electrotransferred to an Immobilon-P membrane (Millipore, Bedford, MA, USA) using a Bio-Rad Trans-Blot SD cell (Bio-Rad Laboratories, Hercules, CA, USA). The membranes were then blocked with 5% milk in PBS-T (PBS containing 0.1% Tween 20) for 1 h at room temperature and incubated in PBS-T containing 5% milk and the relevant primary antibody overnight at 4 °C. After final washing with PBS-T, the membranes were incubated in PBS-T containing goat antimouse or antirabbit antibody HRP conjugates (1:1000, Cell Signaling Technologies, Danvers, MA, USA) for 1 h. The membranes were washed with PBS-T, and signals were visualized using a Clarity Western ECL solution (Bio-Rad) and a Bio-Rad ChemiDoc Touch imager. The digital images of Western blot bands were quantified by densitometric analysis using the Bio-Rad Image Lab (version 5.2.1) software program. The expression levels were normalized to GAPDH and beta-tubulin (TUBB) levels.

### 2.5. Immunofluorescence

For immunofluorescence experiments, pancreatic cancer cells were cultured overnight on 2-well chamber CultureSlides (BD Biosciences, Bedford, MA, USA). Before staining, the cells were washed with cold PBS and fixed with 4% paraformaldehyde in PBS for 30 min at 4 °C. After washing once with PBS, fixed cells were permeabilized in PBS containing 0.5% Triton X-100 for 10 min and then blocked with 10% goat serum (Invitrogen) in PBS at 4 °C. The blocked cells were then incubated with the primary antiserum in PBS containing 10% goat serum for 1 h at room temperature. After further washes with 0.1% Tween 20 in PBS, an Alexa Fluor 555 goat antimouse IgG secondary antibody or Alexa Fluor 555 goat antirabbit IgG (Invitrogen) secondary antibodies were applied for 1 h. After the final wash, slides were incubated for 1 h with DAPI (1 µg/mL in PBS) for nuclei staining and with FITC-labeled antipan cytokeratin (C11) (Sigma-Aldrich) for total cellular cytokeratin stain, washed with PBS, and finally mounted with the ProLong Antifade Reagent (Invitrogen). Slides were observed the next day under a Nikon TE2000U microscope equipped with epifluorescence optics (Nikon Europe, Amsterdam, Netherlands). Images were captured with a cooled CCD camera (DS-5Mc, Nikon).

### 2.6. RNA Isolation, Reverse Transcription, and Quantitative PCR Analysis

Total RNA was extracted using the Extract RNA reagent (Evrogen JSC, Moscow, Russia) and reverse-transcribed into cDNA using Mint reverse transcriptase (Evrogen). qPCR was performed on a LightCycler 480 real-time PCR platform (Roche Applied Science, Mannheim, Germany). qPCR primer sequences are presented in Supplementary Material Table S3. qPCRmix-HSSYBR (Evrogen) was used to determine the relative RNA expression. The PCR reaction conditions were as follows: 1 cycle at 90 °C for 5 min; 40 cycles at 95 °C for 20 s, 60 °C for 20 s, and 72 °C for 35 s; and 1 cycle at 95 °C for 5 s, 55 °C for 60 s, and 97 °C for 15 s. The experiments were performed in triplicate for each sample. Calculations for the relative expression ratio were performed according to Ganger et al. [23]. The results of RT-qPCR are presented as the mean of three independent experiments and normalized against HPRT expression.

### 2.7. RNA-Sequencing (RNA-Seq)

Total RNA from each sample was extracted using an RNeasy Mini Kit (Qiagen GmbH, Hilden, Germany). The quality of the extracted RNA was estimated with an Agilent High Sensitivity RNA ScreenTape using TapeStation 2200 (Agilent Technologies, Santa Clara, CA, USA). PolyA RNA isolation was performed with a NEBNext Poly(A) mRNA Magnetic Isolation Module (New England Biolabs, Ipswich, MA, USA). A NEBNext Ultra II Directional RNA Library Prep Kit for Illumina (New England Biolabs) and a NEBNext Multiplex Oligos for Illumina were used for the preparation of libraries. The preparation of the libraries was performed in biological triplicate for Panc1/siNeg, Panc1/siSOX9, Colo357/siNeg, and Colo357/siSOX9. The sequencing was performed with an Illumina NovaSeq 6000 System (Illumina, San Diego, CA, USA). The quality of raw reads was assessed using the MultiQC tool. The raw reads were mapped on the human reference genome (hg38) using HISAT2 (Galaxy Version 2.1.0 + galaxy 5, usegalaxy.org, accessed on 25 May 2022). The featureCounts (Galaxy Version 1.6.4 + galaxy 1, usegalaxy.org, accessed on 25 May 2022) was used for counting reads to GENCODE release 33 annotated genes [24]. The obtained read counts were converted into transcripts per kilobase million (TPM) values [25].

### 2.8. Cell Proliferation Assays and Cell Cycle Analysis

BxPC-3, Colo357, MiaPaCa-2, and Panc1 cells seeded in 6-well plates were transfected with siSOX9 and control siNeg, as described above. After 72 h, cell proliferation was measured by counting cells using the TC20 automated cell counter (Bio-Rad). MTS assay was performed using the CellTiter 96 AQueous One Solution Cell Proliferation Assay (MTS) (Promega, Madison, WI, USA). An MTS solution (5 mg/mL; 20 µL) was added into each well. The MTS solution was aspirated off following incubation for 1 h at 37 °C. The absorbance of each plate was measured at 595 nm using a Benchmark Plus microplate reader (Bio-Rad). The rates of proliferation siNeg and siSOX9 treated cell was counted relative to the absorbance on the first day of the experiment. For cell cycle analysis, cells collected in the logarithmic phase were fixed with cold 70% ethanol and stained with propidium iodide (PI) staining solution for 30 min. PI fluorescence was analyzed by a Cytomics FC500 (Beckman Coulter, Inc., Brea, CA, USA) flow cytometry system. The distribution of cells into cell cycle stages was determined using a MultiCycle software (Beckman Coulter).

### 2.9. Flow Cytometry Analysis of Apoptosis

Panc1 cells seeded in 6-well plates were transfected with siSOX9 and control siNeg, as described above. After 72 h, cells were trypsinized and suspended in 1 mL of cold PBS. From each cell suspension, 1 × 10^5^ cell was centrifuged and resuspended in 200 μL 1× Annexin V binding buffer (BioLegend, San Diego, CA, USA). In each sample, 10 μL of Annexin V–Alexa Fluor 488 (Invitrogen) and 10 μL of DAPI (1 μg/mL in 1× Annexin V binding buffer) were added, incubated for 15 min at room temperature in dark, and immediately analyzed by flow cytometry (BD FACSAria III cytometer, BD Biosciences, Franklin Lakes, NJ, USA) using the FlowJo v10 software program (BD Biosciences). The Annexin V–AF488-positive and DAPI-negative cells were considered to be early apoptotic cells.

### 2.10. Senescence-Associated β-Galactosidase (SA-β-Gal) Activity Assay

BxPC-3, Colo357, MiaPaCa-2, and Panc1 cells seeded in 6-well plates were transfected with siSOX9 and control siNeg, as described above. After 72 h, SA-β-Gal in cell extracts was measured by the rate of hydrolysis of the nonfluorescent substrate 4-methylumbelliferyl-β-D-galactopyranoside (MUG) to the fluorescent product 4-methylumbelliferone at pH 6.0, as described by Gary et al. [26]. After siRNA transfection, cells were briefly washed twice with PBS and lysed via the addition of 250 µL of CHAPS buffer (5 mM CHAPS, 40 mM citric acid, 40 mM sodium phosphate, and 0.5 mM AEBSF, pH 6.0). Cell lysates were centrifuged for 20 min at 12,000× *g* at 4 °C. The protein concentrations of clarified cell extracts were determined by a Micro BCA Protein Assay Kit (Pierce Biotechnology, Rockford, IL, USA). The clarified lysate (50 µL) was mixed with 50 µL of 2× reaction buffer (40 mM citric acid, 40 mM sodium phosphate, 300 mM NaCl, 10 mM 2-mercaptoethanol, 4 mM MgCl_2_, and 1.7 mM MUG, pH 6.0). After 120 min at 37 °C, 1 mL of 400 mL Na_2_CO_3_ stop solution was added. The carbonate stopped reaction mix was read at 200 µL per well in a black-walled 96-well plate using a Tecan GENios Pro automated plate reader with excitation at 360 nm and emission at 465 nm. The results were divided by the amount of total protein per assay to correctly account for the differences in cell extract protein concentration.

### 2.11. Caspase Activity Assay

BxPC-3, Colo357, MiaPaCa-2, and Panc1 transfected with siSOX9 and control siNeg were seeded at 30 × 10^3^ cells per well into white-walled 96-well plates with complete medium in triplicate. The activity of caspase 3/7, caspase 8, and caspase 9 was determined using Caspase-Glo kits (Promega, Madison, WI, USA), according to the manufacturer’s protocols. Luminescence readings using a Tecan GENios Pro automated plate reader were taken 2 h after adding Caspase-Glo reagents.

### 2.12. Xenotransplantation of Cancer Cells into Danio rerio Embryos

Xenotransplantation of Panc1 cells transfected with siSOX9 and control siNeg was performed using Panc1-EGFP cells. Dechorionized embryos (48 h postfertilization) were injected into the yolk sac using a PicoPump PV820 pneumatic microinjector (World Precision Instruments, Inc., Sarasota, FL, USA), as previously described [27]. The bioimaging of embryos was performed 2 days after transplantation using a Leica ICC50 HD fluorescence microscope (Leica Microsystems CMS GmbH, Wetzlar, Germany) equipped with a GFP filter. Evaluation of Panc1-EGFP cell migration was performed using the digitized images of injected embryos.

### 2.13. Statistical Analysis

The data were analyzed using Prism 6.0 (GraphPad Software, San Diego, CA, USA) and expressed as mean ± standard error of the mean (SEM). Statistical significance was assessed using the Wilcoxon–Mann–Whitney test. For all the tests, *p*-values ≤ 0.05 were considered statistically significant. Asterisks (* and **) indicate statistical significance (*p* ≤ 0.05 and *p* ≤ 0.005, respectively).

## 3. Results

### 3.1. Expression Levels of the SOX9 Protein and RNA in Pancreatic Cancer Cell Lines with Different Levels of Differentiation

**Figure 1:**
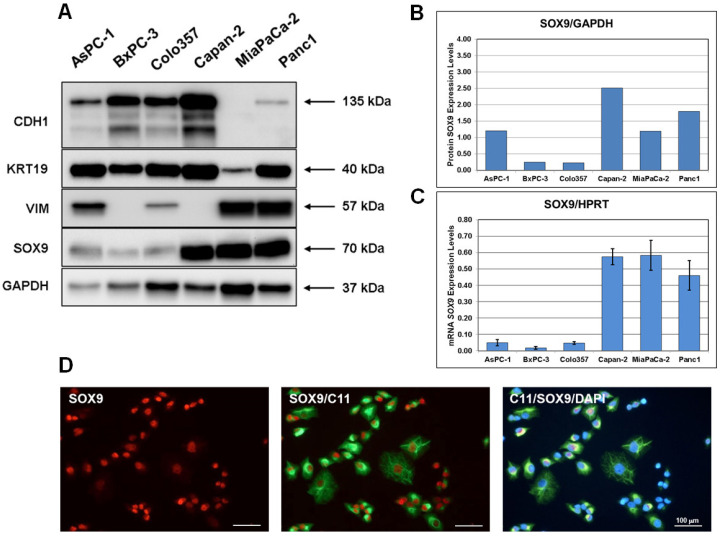
SOX9 protein and *SOX9* mRNA expression in AsPC-1, BxPC-3, Colo357, Capan-2, MiaPaCa-2, and Panc1 cells. (**A**) Western blot analysis of the expression of E-cadherin (CDH1), cytokeratin-19 (KRT19), vimentin (VIM), SOX9, and GAPDH in the investigated cell lines. Pancreatic cancer cells were seeded in 6-well plates (0.5 × 10^6^ cells per well). After 48 h, cell lysates were prepared by adding SDS sample buffer (200 µL per well). (**B**) Densitometric quantitation of Western blots from (**A**). The levels of the SOX9 protein were normalized to the levels of the GAPDH protein. (**C**) Real-time qPCR analysis of the mRNA expression of *SOX9* in pancreatic cancer lines. The results are presented as the mean ± SEM of three independent experiments and normalized against the *HPRT* expression. (**D**) Immunofluorescence imaging of the SOX9 expression in Panc1 cells. Cells were stained for total cytokeratin (green) and SOX9 (red). Nuclei were stained with DAPI (blue). Scale bar = 100 µm.

Prior to studying the effects of SOX9 on differentiation and cell growth in pancreatic cancer, we investigated the expression level of the SOX9 protein in several well-characterized pancreatic cancer cell lines, AsPC-1, BxPC-3, Colo357, Capan-2, MiaPaCa-2, and Panc1 [28] (Figure 1A). Two out of the six cell lines we studied, BxPC-3 and Capan-2, are considered to be moderately and well-differentiated cell types, respectively. These cell lines express the epithelial markers E-cadherin (CDH1) and cytokeratin-19 (KRT19) and do not express the mesenchymal marker vimentin (VIM). The cell lines AsPC-1, Colo357, MiaPaCa-2, and Panc1 are classified as the poorly differentiated quasimesenchymal type of pancreatic cancer cells. These cell lines express the mesenchymal marker vimentin, as well as the epithelial marker E-cadherin, albeit at a relatively low level. The MiaPaCa-2 cell line is characterized by the extremely low expression level of E-cadherin and low expression of cytokeratin-19 (Figure 1A). The SOX9 protein had different expression levels in analyzed cell lines (Figure 1B). The cell lines BxPC-3 and Colo357 were characterized by a low level of SOX9 protein expression, while the AsPC-1, Capan-2, MiaPaCa-2, and Panc1 cell lines demonstrated a relatively high expression level of the SOX9 protein. The expression levels of *SOX9* RNA determined by RT-qPCR in the investigated cell lines corresponded with the relative levels of the SOX9 protein, with the exception of AsPC-1 cells, which demonstrated a lowered level of *SOX9* RNA (Figure 1C). This may be due to the high stability of the SOX9 protein in AsPC-1 cells. In all the investigated cell lines, SOX9 demonstrated predominantly nuclear localization (Figure 1D and Figure S1).

### 3.2. The Cell-Specific Effect of SOX9 on the Expression Levels of Protein Markers of Pancreatic Cancer Differentiation

**Figure 2:**
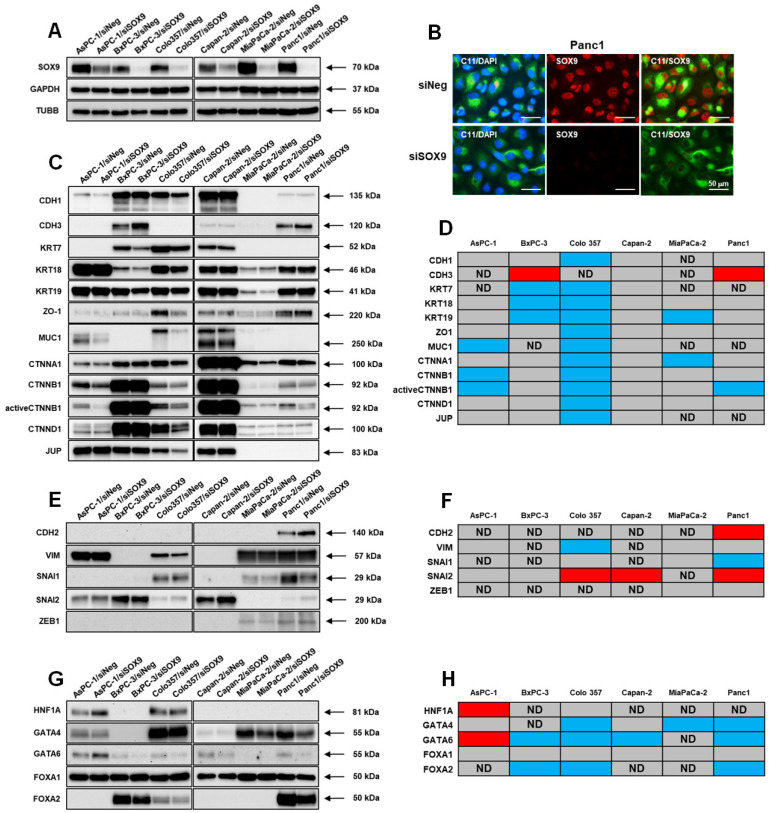
The effect of SOX9 downregulation on the expression levels of protein markers of differentiation and developmental transcription factors in pancreatic cancer cells. (**A**) Western blot analysis of the expression of SOX9 in AsPC-1, BxPC-3, Colo357, Capan-2, MiaPaCa-2, and Panc1 cells transfected with control siNeg and siSOX9 (*n* = 3). GAPDH and beta-tubulin (TUBB) were used as loading and normalization controls. (**B**) Immunofluorescence imaging of the SOX9 expression in Panc1 cells transfected with control siNeg and siSOX9. Cells were stained for total cytokeratin (green) and SOX9 (red). Nuclei were stained with DAPI (blue). Scale bar = 50 µm. (**C**) Western blot analysis of the expression of epithelial cell protein markers in the investigated cell lines transfected with control siNeg and siSOX9 (*n* = 3). (**D**) Heatmap of differentially expressed epithelial cell proteins from (**C**). Color code: red, upregulation; blue, downregulation; gray, no change. ND = undetected expression. (**E**) Western blot analysis of the expression of mesenchymal cell protein markers in the investigated cell lines transfected with control siNeg and siSOX9 (*n* = 3). (**F**) Heatmap of differentially expressed mesenchymal cell proteins from (**E**). Color code: red, upregulation; blue, downregulation; gray, no change. ND = undetected expression. (**G**) Western blot analysis of the expression of developmental transcription factors in the investigated cell lines transfected with control siNeg and siSOX9 (*n* = 3). (**H**) Heatmap of differentially expressed developmental transcription factors from (**G**). Color code: red, upregulation; blue, downregulation; gray, no change. ND = undetected expression.

For the purpose of studying the effect of the SOX9 protein expression on the pancreatic tumor phenotype, we chose to downregulate the SOX9 expression via synthetic siRNA targeting SOX9 RNA. Three different siRNAs were synthesized and tested by transient transfection into Panc1 cells. These preliminary experiments demonstrated that the equimolar mix of all three siRNAs had the greatest suppressive effect (Supplementary Material Figure S2A,B). This mix of three siRNAs (siSOX9) was therefore used in all subsequent experiments. The transfection did not lead to significant changes in the expression levels of the two housekeeping proteins that were later used as internal normalization controls in our subsequent experiments (GAPDH and TUBB). It is worthy of note that in the case of Panc1 cells, the decrease in SOX9 expression levels led to significant changes in cell morphology (Supplementary Material Figure S2C). siSOX9 transfection effectively downregulated the expression of SOX9 in all the investigated pancreatic cancer cell lines (Figure 2A). The strongest downregulation level of SOX9 was achieved in the cell lines BxPC-3, Colo357, MiaPaCa-2, and Panc1, according to Western blot analysis (Supplementary Material Figure S3A). Additionally, in Panc1 cells transfected with siSOX9, the SOX9 protein was detected via immunofluorescent analysis at a significantly lower level, as compared with control cells transfected with siNeg (Figure 2B).

Several previous studies have shown that the increased expression of SOX9 in human tumor tissue may lead to a decrease in epithelial differentiation of cancer cells and the development of tumor-associated epithelial–mesenchymal transition (EMT) [29,30,31,32,33]. In our experiments, the downregulation of SOX9 in siSOX9 transfected cells induced a decrease in the expression of an important epithelial marker, CDH1, only in the Colo357 cell line (Figure 2C,D). The level of CDH1 expression in the cell lines AsPC-1, BxPC-3, Capan-2, and Panc1 remained unchanged (Supplementary Material Figure S3C). At the same time, the expression of the epithelial marker CDH3 (P-cadherin) increased in the cell lines BxPC-3 and Panc1. The epithelial cytokeratins KRT7, KRT18, and KRT19 had coordinately lowered expression in the cell lines BxPC-3 and Colo357 (Supplementary Material Figure S3C). A significant decrease in the levels of KRT19 was detected in cells of the poorly differentiated line MiaPaCa-2. The expression of the epithelial cell marker tight junction protein ZO-1 was significantly decreased only in siSOX9 transfected Colo357 cells, as compared with control cells. The expression of MUC1 decreased in the siSOX9 transfected cell lines AsPC-1 and Colo357, while in the Capan-2 cell line, it remained unchanged (Supplementary Material Figure S3C). The expression levels of proteins, associated with intercellular contacts between epithelial cells, α-E-catenin, CTNNB1 (β-catenin), CTNND1 (δ-1-catenin), and JUP (γ-catenin), decreased in the siSOX9 transfected cell line Colo357. The levels of the CTNNA1 protein also noticeably decreased after the transfection of siSOX9 into MiaPaCa-2 cells. For a number of tumors, the protein SOX9 has been shown to be a potential regulator of the activity of the Wnt/β-Catenin signaling pathway [31,32]. Interestingly, in our experiments, transfection of siSOX9 into AsPC-1 and Colo357 cells led to a decrease in total and active (nonphosphorylated at Ser45) levels of the β-catenin protein. The downregulation of SOX9 expression levels in Panc1 did not lead to a decrease in total β-catenin, but did induce a decrease in active nonphosphorylated β-catenin. Thus, the downregulation of the SOX9 expression did not lead to the expected enhancement of the epithelial status of the cells of the pancreatic cancer lines studied.

We also investigated the effect of SOX9 downregulation on the expression of mesenchymal phenotype markers and EMT regulators (Figure 2E,F and Figure S3E). CDH2 (N-cadherin), the protein present in intercellular junctions of mesenchymal phenotype cells, was detected via Western blot analysis only in Panc1 cells. The expression level of N-cadherin in Panc1 cells increased noticeably after siSOX9 transfection. The level of expression of vimentin, the intermediate filament protein of mesenchymal cells, was lowered in siSOX9 transfected Colo357 cells. Interestingly, in Panc1 cells with downregulated SOX9 expression, we discovered a reliable decrease in the level of SNAI1, an important transcription factor involved in EMT. However, the expression of another member of the Snail factor family, SNAI2 (Slug), significantly increased in the siSOX9 transfected cell lines Colo357, Capan-2, and Panc1 and did not change in the cell lines AsPC-1 and BxPC-3. The level of expression of another EMT-related transcription factor, ZEB1, did not change in pancreatic cancer cell lines after the downregulation of SOX9. Therefore, the downregulation of SOX9 expression levels in the investigated pancreatic cancer cell lines induced specific and multidirectional changes in the expression of the markers and regulators of mesenchymal and epithelial differentiation in a cell-specific manner.

### 3.3. SOX9 and the Expression of Developmental Transcription Factors in Pancreatic Cancer Cells

During the development of pancreas, SOX9 plays a key role as one of the transcription factors whose coordinated interactions in early precursor cells drive the pancreatic organogenesis process [19]. Some developmental transcription factors are differentially expressed in pancreatic tumors as well, and they take part in the formation of unique tumor phenotypes [34,35,36,37]. Therefore, we performed Western blot analysis of the HNF1A, GATA4, GATA6, FOXA1, and FOXA2 proteins in pancreatic cancer cell lines with downregulated SOX9 expression for the purpose of investigating the potential functional link between SOX9 and the expression of these protein master regulators of pancreatic development (Figure 2G,H and Figure S3G). The HNF1A protein was only detected in the cell lines AsPC-1 and Colo357. The downregulation of SOX9 led to a detectable increase in the expression of HNF1A in AsPC-1 cells and did not have an effect on the HNF1A expression in Colo357 cells. The expression levels of the transcription factor GATA4 decreased in the siSOX9 transfected cell lines Colo357, MiaPaCa-2, and Panc1, and in the cell lines AsPC-1 and Capan-2, the levels of GATA4 remained unchanged. The expression of GATA6, a transcription factor from the same protein family as GATA4, was lowered in the cell lines BxPC-3, Colo357, Capan-2, and Panc1. Interestingly, in AsPC-1 cells with downregulated SOX9 expression, the expression levels of GATA6 increased, as compared with control cells. The downregulation of SOX9 did not lead to detectable changes in the expression of the transcription factor FOXA1 in all the six investigated pancreatic cancer cell lines. At the same time, in the cell lines BxPC-3, Colo357, and Panc1, the level of expression of another transcription factor, FOXA2, decreased after the transfection of siSOX9. It should also be noted that we did not detect the expression of the PDX1 and PTF1A proteins in cell lysates of all the six pancreatic cancer lines analyzed (data not shown). Therefore, our results demonstrate that the suppression of SOX9 expression in pancreatic cancer cells leads to specific changes in the expression profiles of development regulatory proteins.

### 3.4. The Downregulation of SOX9 Affects the Expression of Cell Cycle Proteins

**Figure 3:**
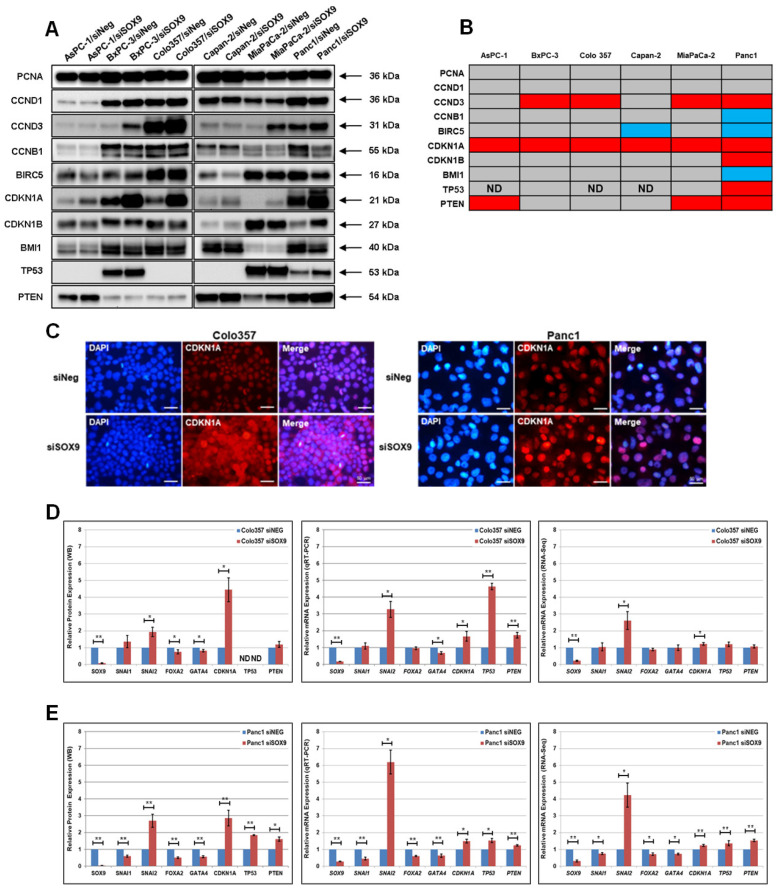
The effect of SOX9 downregulation on the expression levels of cell cycle protein regulators. (**A**) Western blot analysis of the expression of cell cycle protein regulators in the investigated cell lines transfected with control siNeg and siSOX9 (*n* = 3). (**B**) Heatmap of differentially expressed cell cycle protein regulators from (**B**). Color code: red, upregulation; blue, downregulation; gray, no change. ND = undetected expression. (**C**) Immunofluorescence imaging of p21 Waf1/Cip1 (CDKN1A) expression in Colo357 and Panc1 cells transfected with control siNeg and siSOX9. Cells were stained for SOX9 (red). Nuclei were stained with DAPI (blue). Scale bar = 50 μm. (**D**,**E**) Relative Western blot, RT-qPCR, and RNA-Seq data quantification of the levels of SOX9, SNAI1, SNAI2, FOXA2, GATA4, CDKN1A, TP53, and PTEN in control siNeg and siSOX9 transfected Colo357 (**D**) and Panc1 (**E**) cells. RT-qPCR and RNA-Seq data are means ± SEM from three technical replicates and representative of at least three experiments. * *p* ≤ 0.05; ** *p* ≤ 0.01 compared with siNeg control.

As a rule, the elevated SOX9 expression in cancer cells leads to the stimulation of cell proliferation [38,39], but for a number of tumors, it has been shown that increased SOX9 expression can inhibit cell growth and division [15,16,17]. We analyzed the effect of SOX9 downregulation on the expression of some cell cycle proteins and protein inhibitors of cell division (Figure 3A–C and Figure S4A). It was rather unexpected that the downregulation of SOX9 in all the six investigated pancreatic cancer cell lines did not significantly alter the expression of cyclin D1 (CCND1) and PCNA. At the same time, during the downregulation of SOX9 in the cell lines BxPC-3, Colo357, MiaPaCa-2, and Panc1, the expression level of cyclin D3 (CCND3) increased significantly, as compared with control cells. Additionally, the downregulation of SOX9 in Panc1 cells led to a pronounced decrease in the expression levels of cyclin B1 (CCNB1). In the cell lines Capan-2 and Panc1, the transfection of siSOX9 induced a decrease in the expression levels of the regulator of the cell division and apoptosis, survivin (BIRC5). In all the investigated pancreatic cancer cell lines, the downregulation of SOX9 led to an increase in the levels of the cell cycle inhibitor protein (CDKN1A). We have demonstrated, using the immunofluorescence method, that in Panc1 cells transfected with siSOX9, the expression levels of nuclear p21Waf1/Cip1 increased. Additionally, in Colo357 cells with downregulated SOX9 expression, the levels of cytoplasmic CDKN1A increased as well (Figure 3C). The transfection of siSOX9 into Panc1 cells led to an increase in the expression of another cell cycle inhibitor, p27Kip1 (CDKN1B). In other cell lines with downregulated SOX9, the level of p27Kip1 did not change. We also investigated the expression of two well-known tumor suppressors and regulators of cell cycle progression, TP53 and PTEN, in siSOX9 transfected cell lines. In Panc1 cells with experimentally downregulated SOX9, the levels of the tumor suppressor TP53 were elevated. In the cell lines AsPC-1, MiaPaCa-2, and Panc1, the transfection of siSOX9 led to an increase in the expression of the protein PTEN. Additionally, out of all the six investigated cell lines with downregulated SOX9, only Panc1 cells had decreased expression levels of BMI1, a negative regulator of CDKN1A and PTEN transcription. Thus, SOX9 suppression leads to cell-line-specific changes in cell cycle protein expression.

### 3.5. The Downregulation of the SOX9 Protein Affects the Transcriptional Activity of SNAI2, GATA4, CDK1A, TP53, and PTEN Genes in Pancreatic Cancer Cells

Next, we investigated the levels of *SNAI1*, *SNAI2*, *FOXA2*, *GATA4*, *CDKN1A*, *PTEN*, and *TP53* RNA expression in two cell lines, Colo357 and Panc1, with downregulated SOX9 expression, as compared with control cells. The levels of RNA expression were determined via quantitative RT-PCR (qRT-PCR) and the analysis of transcriptome data acquired by RNA-Seq. The results shown in Figure 3D,E demonstrate that the majority of the genes that we studied in both cell lines with downregulated SOX9 expression had a change in the RNA expression levels. For instance, in Colo357 cells, the downregulation of SOX9 levels led to an increase in transcription levels of the *SNAI2* and *CDKN1A* genes, which is consistent with our results on the cells with downregulated SOX9 expression, which was only detectable via qRT-PCR. Interestingly, we discovered an elevated level of *PTEN* gene activity in Colo357 cells after the transfection of siSOX9, which did not correspond with our RNA-Seq and Western blot results. The expression of the TP53 protein was not detected in either control or siSOX9 transfected Colo357 cells, which is likely due to the low-level protein expression of short-lived wild-type TP53 in this cell line. On the other hand, qRT-PCR did show a reliable increase in *TP53* RNA expression in Colo357 cells with downregulated SOX9 expression. At the same time, RNA-Seq data analysis did not detect a significant increase in *TP53* expression. Similar to Colo357 cells, the decrease in SOX9 protein expression in Panc1 cells led to an increase in the transcription levels of *SNAI2* and *CDKN1A*, which correlated with our results demonstrating the changes in SNAI2 and CDKN1A protein levels in those cells. In Panc1 cells with downregulated SOX9 expression genes, *SNAI1*, *FOXA2*, and *GATA4* demonstrated a decrease in transcriptional activity, which correlated with the detected decrease in the expression of the SNAI1, FOXA2, and GATA4 proteins. The experimental downregulation of the SOX9 expression in Panc1 cells led to an increase in the transcriptional activity of the *TP53* and *PTEN* genes, which corresponds with the results of the Western blot analysis of the TP53 and PTEN proteins. Thus, the acquired results of qRT-PCR and RNA-Seq analysis of the transcriptional activity of the SNAI2, GATA4, CDKN1A, TP53, and PTEN genes demonstrated their SOX9-dependent transcriptional regulation in the investigated cell lines, Colo357 and Panc1.

### 3.6. SOX9 Regulates Apoptosis and Affects the Migratory Potential of Pancreatic Cancer Cells

**Figure 4:**
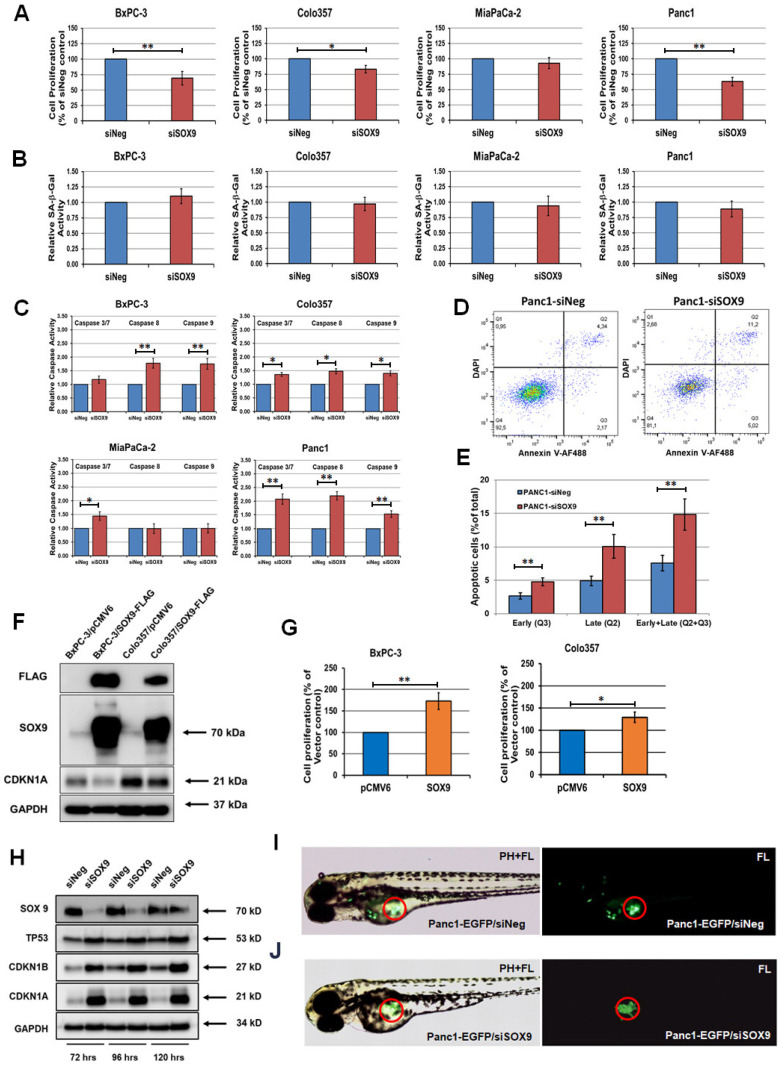
Effect of SOX9 expression on cell proliferation, cellular senescence, apoptosis, and cell migration. (**A**) Effect of SOX9 downregulation on the cell proliferation of BxPC-3, Colo357, MiaPaCa-2, and Panc1. Pancreatic cancer cells seeded in 6-well plates were transfected with siSOX9 and control siNeg. After 72 h, the cell number was measured by counting cells using the TC20 automated cell counter. Data were expressed as the percentage from control cell growth. Data are means ± SEM from three technical replicates and representative of at least three experiments. * *p* ≤ 0.05; ** *p* ≤ 0.01 compared with siNeg control. (**B**) Effect of SOX9 downregulation on the senescence-associated β-galactosidase (SA-β-Gal) activity of BxPC-3, Colo357, MiaPaCa-2, and Panc1 cells. SA-β-Gal activity data were expressed relative to SA-β-Gal activity levels in siNeg and siSOX9 transfected cells. (**C**) Effect of SOX9 downregulation on caspase 3/7, caspase 8, and caspase 9 activities of BxPC-3, Colo357, MiaPaCa-2, and Panc1 cells. Caspase activity data were expressed relative to caspase activity levels in siNeg and siSOX9 transfected cells, respectively. (**D**) Panc1 cells were transfected with siSOX9 and siNeg for 72 h and then analyzed by annexin V–AF488/DAPI staining with flow cytometry analysis. The lower right area (Q3) shows early apoptotic cells, and the upper right area (Q2) shows late apoptotic cells. (**E**) Summary graphs of the flow cytometry results. (**F**) Western blot analysis of SOX9 and CDKN1A expression in Panc1 cells transfected with pCMV6 empty plasmid and pCMV6-SOX9-FLAG after 72 h post-transfection. GAPDH was used as control. (**G**) Effect of SOX9 upregulation on cell proliferation of BxPC-3 and Colo357. Pancreatic cancer cells seeded in 6-well plates were transfected with empty pCMV6 vector and pCMV6-SOX9-FLAG. After 72 h, the cell number was measured by counting cells using the TC20 automated cell counter. (**H**) Western blot analysis of the expression of SOX9, TP53, CDKN1A, and CDKN1B in Panc1 cells transfected with control siNeg and siSOX9. Cell lysates were prepared at 72, 96, and 120 h after first siRNA transfection. GAPDH was used as loading control. (**I**) The representative images displayed the GFP distributions of transplanted Panc1-EGFP control siNeg transfected cells in *Danio rerio* embryo. The microinjection site *Danio rerio* embryo is indicated with a red circle. The bioimaging of injected embryos was performed 2 days after cell transplantation. PH, phase contrast; FL, fluorescence. (**J**) The representative image displayed the GFP distributions of transplanted Panc1-EGFP siSOX9 transfected cells in *Danio rerio* embryo. The microinjection site *Danio rerio* embryo is indicated with a red circle. The bioimaging of injected embryos was performed 2 days after cell transplantation. PH, phase contrast; FL, fluorescence.

The elevated expression of the cell cycle inhibitor protein CDKN1A and tumor suppressors TP53 and PTEN may indicate the induction of apoptosis or cellular senescence in cells with experimentally downregulated SOX9 expression. Initially, we investigated the effect of the suppression of SOX9 expression on BxPC-3, Colo357, MiaPaCa-2, and Panc1 cell lines and on their proliferative activity. A decrease in SOX9 expression caused a considerable inhibitory effect on the proliferation in the BxPC-3, Colo357, and Panc1 cell lines (Figure 4A and Figure S5). At the same time, in MiaPaCa-2 cells, the experimental downregulation in SOX9 expression only led to an insignificant decrease in proliferative activity (Figure 4A). In addition, we investigated the activity of the cell senescence marker SA-β-Gal in lysates of the cell lines BxPC-3, Colo357, MiaPaCa-2, and Panc1 with downregulated SOX9 expression. As shown on Figure 4B, the transfection of siSOX9 into those cell lines did not lead to an increase in SA-β-Gal activity in the cells of all the four lines with suppressed SOX9 expression. In order to analyze the effect of SOX9 expression on cell viability, we measured the levels of proapoptotic caspase activity in the BxPC-3, Colo357, MiaPaCa-2, and Panc1 cell lines with experimental downregulation of SOX9 (Figure 4C). We discovered that 72 h after the first transfection in the siSOX9-transfected BxPC-3, Colo357, and Panc1 cells, the levels of caspase 3/7, caspase 8, and caspase 9 activity were significantly increased, compared with siNeg transfected controls. Additionally, 72 h after the first siSOX9 transfection, we detected an increased level of caspase 3/7 activity in MiaPaCa-2 cells, while the levels of caspase 8 and caspase 9 stayed the same. Additionally, in Panc1 cells, the transfection of siSOX9 leads to a more than twofold increase in the percentage of apoptotic cells (annexin^+^ and annexin^+^/DAPI^+^ cells), compared with siNeg transfected controls (Figure 4D,E).

Additionally, we studied the effect of SOX9 overexpression on cell proliferation on BxPC-3 and Colo357 cells, which are characterized by a relatively low level of SOX protein expression (Figure 4F). The transient transfection of the SOX9-containing expression plasmid led to an accelerated proliferation rate in transfected cells (Figure 4G). In transfected cells, the overexpression of SOX9 was accompanied by a decrease in CDKN1A expression (Figure 4F), which correlates with our acquired results.

We also measured the extent of SOX9 expression downregulation that can be achieved via transient transfection of siSOX9. For this purpose, we analyzed the levels of SOX9 expression 3, 4, and 5 days after the first siSOX9 transfection (Figure 4H). The acquired results demonstrate that the decreased level of SOX9 in Panc1 cells was sustained up to 120 h after the start of the transfection. Additionally, siSOX9-transfected Panc1 cells maintained an increased expression level of TP53, CDKN1A, and CDKN1B up to 120 h after the transfection (Figure 4H). The presence of the extended expression downregulation by siSOX9 allowed us to investigate how the downregulation of SOX9 may affect the mobility and migratory potential of pancreatic cancer cells after their xenotransplantation into *Danio rerio* embryos (Figure 4I,J). For this purpose, stably EGFP-marked Panc1 cells were transfected with siSOX9 and siNeg. Cells were injected into *Danio rerio* embryos 72 h after transfection, and another 24–48 h later, the number of migrated cells was counted. The results of two independent experiments demonstrated that the downregulation of SOX9 decreased the migratory activity of Panc1 cells transplanted into *Danio rerio* embryos (Supplementary Material Table S4). In the control groups, 91 embryos were analyzed, among which 9 (10%) embryos with migrated EGFP-Panc1 cells were identified. At the same time, in the experimental groups of siSOX9 transfected cells, only 1 out of 89 embryos was found with migrated EGFP-Panc1 cells. Therefore, we can conclude that in Panc1 cells, the downregulation of SOX9 leads to decreased proliferative activity, induction of apoptosis, and inhibition of migratory activity.

## 4. Discussion

It is becoming increasingly clear that genes regulating development also play critical roles in cancer initiation and progression [40]. *SOX9* is a developmentally regulated gene required for lineage commitment in different tissues and organs. In normal pancreas, SOX9 maintains the progenitor pool for multiple pancreatic cells, including exocrine and endocrine cells. In pancreatic diseases, SOX9 accelerates acinar-to-ductal metaplasia and has a critical role.

In PanIN formation [8,9], it has been frequently noted that pancreatic cancer is characterized by the high degree of genetic and phenotypic heterogeneity of cells that comprise the tumor [20,41]. The role of SOX9 in the formation and maintenance of various pancreatic tumor cell phenotypes has not been thoroughly studied. In our research, we studied several pancreatic cancer cells lines, which displayed varying levels of differentiation and a range of oncogenic mutations. We discovered that the downregulation of SOX9 expression in the described cell lines leads to cell-line-specific changes in the expression levels of epithelial and mesenchymal protein markers. Additionally, the downregulation of SOX9 expression had a cell-specific effect on the expression of pancreatic developmental master genes. SOX9 downregulation had the greatest effect on the expression levels of the protein regulators of cell proliferation. In three of the four pancreatic cancer cell lines studied, the transfection of siSOX9 led to a significant decrease in cell proliferation and to the activation of proapoptotic caspases in transfected cells.

Previous studies have shown that in a number of cancer types, SOX9 can function as an activator of EMT in tumor cells [14,31,33]. Considering the natural heterogeneity of pancreatic cancer cells, we analyzed the cell response to the downregulation of SOX9 in six different cell lines. Surprisingly, in the analyzed AsPC-1, BxPC-3, and Colo357 cells, the downregulation of SOX9 led to a decrease in the expression of epithelial identity cell markers (CDH1, KRT7, KRT17, KRT19, ZO-1, and MUC1). At the same time, in Capan-2 and Panc1 cells, a similar tendency has not been detected. It is important to note that in the three investigated cell lines of the quasimesenchymal type (AsPC-1, MiaPaCa-2, and Panc1), the downregulation of SOX9 did not affect the levels of expression of the traditional mesenchymal marker vimentin. Additionally, in Panc1 cells, we detected a decrease in the expression levels of the EMT regulator SNAI1 and a simultaneous increase in the levels of the mesenchymal cadherin CDH2. Additionally, in the cell lines Colo357, Capan-2, and Panc1, the expression of another EMT factor, SNAI2, increased in response to SOX9 downregulation, which demonstrated the negative regulation of its protein level by SOX9. In addition to our experimental results, we also analyzed the available data from the Cancer Genome Atlas (TCGA) to see whether there is any correlation between the expression of SOX9 and the expression of epithelial phenotype marker genes (CDH1, KRT7, KRT18, and KRT19) and two markers of the mesenchymal phenotype (CDH2 and VIM). The acquired data do not allow us to verify the role of SOX9 as the regulator of EMT and the activator of mesenchymal differentiation genes in pancreatic tumors. For instance, in pancreatic tumors, the expression of SOX9 positively correlates with the expression of epithelial phenotype genes (CDH1, KRT7, KRT18, and KRT19) and negatively correlates with the expression of the mesenchymal phenotype gene VIM (Supplementary Material Figure S6A). Additionally, the analysis of 29 pancreatic cancer cell lines did not show any definitive correlations between SOX9 expression and the expression of those genes either (Supplementary Material Figure S6B). To evaluate the expression levels of the SOX9, CDH1, KRT7, KRT18, KRT19, CDH2, and VIM genes in pancreatic cancer cell lines, the RNA-Seq data [42] from the Expression Atlas (www.ebi.ac.uk/gxa/home (accessed on 13 April 2022)) have been used. In our opinion, the acquired results do not allow us to consider SOX9 as a factor that stimulates EMT in PDAC cells. This hypothesis is indirectly supported by the results of our work, which demonstrate that TGF-β1-induced EMT in Panc1 cells coincides with a decrease in SOX9 RNA expression [43].

Interesting results were acquired while studying the expression of several transcription factors of pancreatic embryonic development in cells with downregulated SOX9 expression. Similar to the case with differentiation markers, we observed cell-line-specific changes in expression levels. In our study, we discovered a link between the expression of SOX9 in pancreatic cancer cell lines and the expression of the GATA4 and GATA6 proteins. The GATA proteins are well-known structurally related zinc finger transcription factors that bind to DNA at the consensus GATA motif sequence. GATA factors are important for cell fate determination. GATA4 and GATA6 play roles in the endoderm formation and differentiation into respiratory tract epithelium and digestive tract. These two GATA transcription factors are increasingly recognized as key players in pancreatic carcinogenesis [35,36]. In our experiments, the GATA4 factor demonstrated a decrease in the expression in three out of six pancreatic cancer cell lines (Colo357, MiaPaCa-2, and Panc1) with the downregulated expression of SOX9. It should be noted that in PDAC cells, GATA4 plays a role as a differentiation factor and also functions as a tumor suppressor. In glioblastoma cells, an increase in GATA4 levels can lead to TP53-independent upregulation of CDKN1A and cell growth suppression [44]. In BxPC-3, Colo357, Capan-2, and Panc1 cells with downregulated SOX9 protein expression, the levels of GATA6 also decreased, which may suggest that SOX9 takes part in the regulation of GATA6 expression in these cell lines. Surprisingly, the downregulation of SOX9 in the AsPC-1 cell line led to a significant elevation in GATA6 expression levels.

Earlier, it has been shown for a group of tumor types that the SOX9 factor affects cancer cell division, since SOX9 overexpression increases the proliferation of a group of cell lines, and the downregulation of SOX9 expression slows down the proliferation [12,13,14]. In our experiments, we also showed that BxPC-3, Colo357, and Panc1 cells with downregulated SOX9 expression had lower proliferative activity than control cells. At the same time, the decrease in SOX9 expression did not lead to the inhibition of proliferation in MiaPaCa-2 cells. It is important to note that after the downregulation of SOX9 in pancreatic cancer cells, the expression levels of two oncosuppressors, PTEN and TP53, were elevated. In the cell lines AsPC-1, MiaPaCa-2, and Panc1, the levels of the detectable protein PTEN were increased, and in Panc1 cells, the levels of TP53 were significantly elevated as well. Besides that, the level of *TP53* RNA increased in Colo357 cells after the downregulation of SOX9. Usually, the elevated expression of PTEN and TP53 leads to the suppression of cell division, which can be the reason behind the antiproliferative effect of SOX9 downregulation in the investigated cell lines. In all the cell lines we investigated, the downregulation of SOX9 led to an increase in the amount of the CDKN1A protein, the elevated expression of which usually inhibits cell proliferation and, in some cases, may induce cellular senescence. The functional link between the expression of SOX9 and CDKN1A was demonstrated on a group of human tumors. For example, Jiang et al. showed that in the cells of lung adenocarcinoma, SOX9 is a negative regulator of CDKN1A, and the downregulation of SOX9 leads to an increased expression of CDKN1A and to the inhibition of cell proliferation [13]. It has also been recently shown for cells of several types of cancer (glioma, pancreatic cancer, and stomach cancer) that the elevated expression of SOX9 leads to a decrease in CDKN1A and to the stimulation of cell division [45]. In our work, we also showed that the overexpression of SOX9 in two pancreatic cancer cell lines, BxPC-3 and Colo357, results in increased cell proliferative activity and decreased CDKN1A expression levels. Considering these data, the results we acquired also suggest that in pancreatic cancer cells, SOX9 is a negative regulator of CDKN1A activity. In connection with this, it can be noted that in Panc1 cells with downregulated SOX9, the expression level of BMI1, a well-known transcription inhibitor of CDKN1B and PTEN, was lowered [45,46]. One of the more known activators of the CDKN1A gene is the tumor suppressor protein TP53 [47]. However, only two out of the six investigated cell lines, Capan-2 and Colo357, contain wild-type TP53 and are capable of TP53-dependent activation of the CDKN1A promoter. The cell line AsPC-1 is characterized by the heterozygote mutation of TP53 (p.C135fs*35), BxPC-3 cells contain the p.Y220C mutation of TP53, MiaPaCa-2 cells contain the p.R248W mutation of TP53, and Panc1 cells contain the p.R273H mutation of TP53 [28,48,49]. At present, we do not know whether the mentioned mutated forms of TP53 are capable of transactivating the CDKN1A gene promoter in the investigated cell lines. The answer to this question requires additional experimental research. It is also worthy of note that Capan-2 and Panc1 cells with downregulated SOX9 expression displayed a decrease in the expression of the antiapoptotic protein BIRC5, whose gene is regulated by TP53 [50].

An important result of our work is that the suppression of SOX9 in three cell lines, BxPC-3, Colo357, and Panc1, not only leads to the inhibition of cell proliferation but also activates apoptotic caspases in these cells. In addition, the downregulation of SOX9 in Panc1 cells leads to a marked suppression of cell migration after their transplantation into *Danio rerio* embryos. At the same time, due to certain limitations of the *Danio rerio* organismal model, the obtained results require independent confirmatory experiments in other in vivo models of tumor metastasis. Thus, our results demonstrate the possibility of viewing SOX9 as a potential target for antitumor therapy for pancreatic cancer.

## 5. Conclusions

In summary, despite the fact that our research was not aimed at describing the full scope of molecular and physiological changes in various pancreatic cancer lines with downregulated SOX9, the acquired results allow us to draw the following important conclusions: First, the transcription factor SOX9 executes multilevel control over important intracellular processes, which involve the maintenance of cell differentiation, the regulation of cell division, and apoptosis. Second, we conclude that cell response to SOX9 expression downregulation depends on the cell line and is apparently defined by the genomic and epigenomic changes in pancreatic cancer cells, which were acquired during carcinogenesis. Third, the therapeutic targeting of the activity and expression of the SOX9 protein, despite its multiple effects on intracellular activities, may slow down proliferation and induce apoptosis in pancreatic cancer cells.

## Data Availability

The data presented in this study are available on request from the corresponding author.

## Supplementary Materials

The following supporting information can be downloaded at: https://www.mdpi.com/article/10.3390/biomedicines10071466/s1. Figure S1: Immunofluorescence imaging of SOX9 expression in AsPC-1, BxPC-3, Colo357, Capan-2, MiaPaCa-2 and Panc1 cells; Figure S2: (A) Western blot analysis of SOX9 expression in Panc1 cells transfected with control siNeg, three different siRNAs and their equimolar mix (siSOX9). GAPDH was used as loading and normalization control. (B) Densitometric quantitation of Western blots from SF2A. (C) Morphological changes in Panc1 cancer cells induced by siSOX9 transfection. Phase contrast. Scale bar = 100 μm; Figure S3: Densitometric quantitation of Western blots from Figure 2A,C,E,G (*n* = 3); Figure S4: Densitometric quantitation of Western blots from Figure 3A (*n* = 3); Figure S5: The effect of SOX9 downregulation on Panc1 cell proliferation; Figure S6: Expression of SOX9 and EMT markers in pancreatic cancer tumors and cell lines. Table S1: List of the antibodies used for Western Blots and immunofluorescence imaging; Table S2: List of the siRNAs used for targeting human SOX9 RNA; Table S3: List of the primers used for RT-qPCR; Table S4: Results of two independent experiments demonstrated that the downregulation of SOX9 decreased the migratory activity of Panc1-EGFP cells transplanted into *Danio rerio* embryos.
