# Supplementary material for: SOX9 Protein in Pancreatic Cancer Regulates Multiple Cellular Networks in a Cell-Specific Manner
Source: Biomedicines. 2022 Jun 21;10(7):1466. doi: 10.3390/biomedicines10071466 (PMC9312990; doi:10.3390/biomedicines10071466)
Supplement: Supplementary file 1 [file biomedicines-10-01466-s001.zip › biomedicines-1766456-supplementary proof/Figure S4.pdf]

A

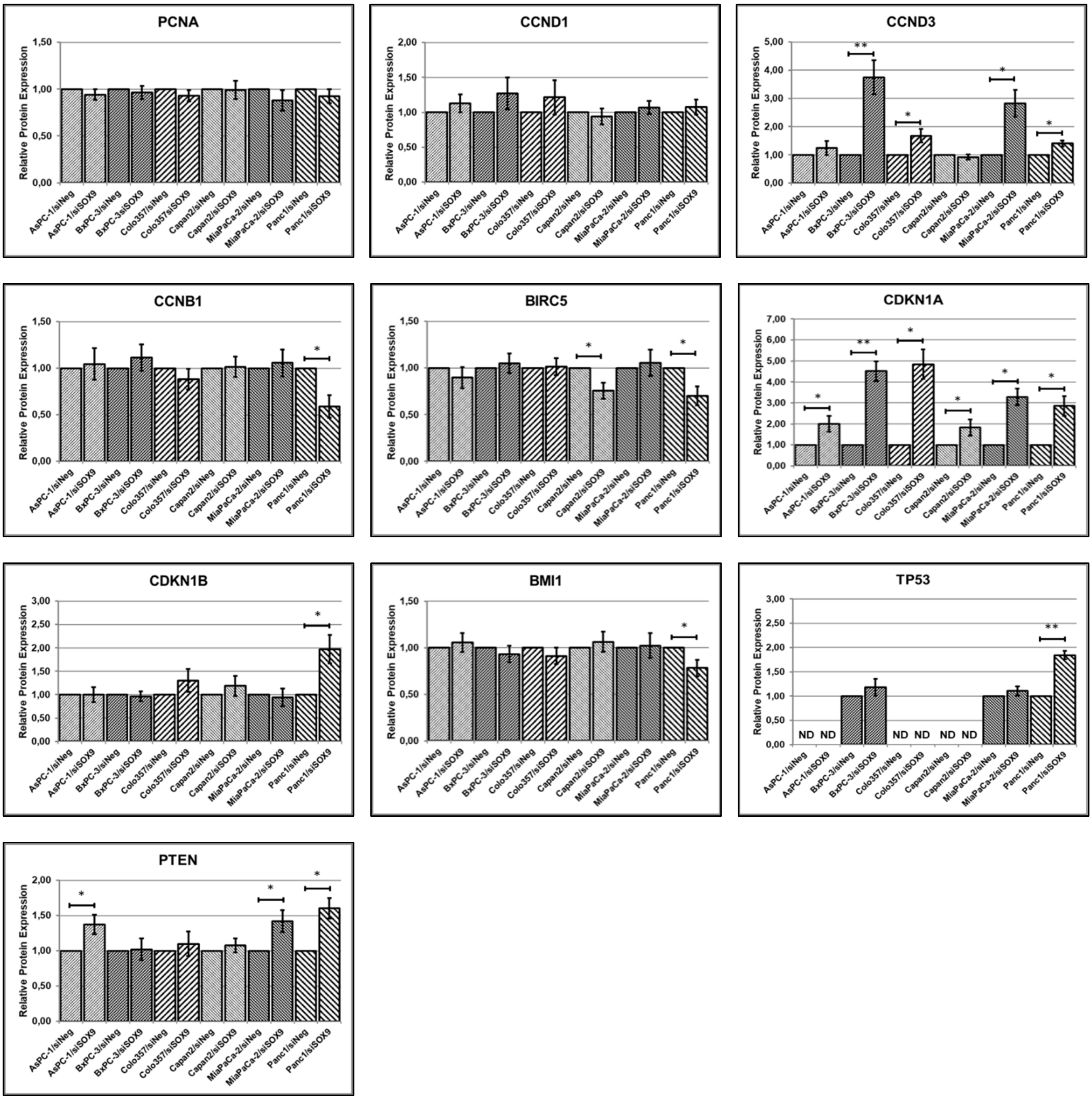

Figure S4. Densitometric quantitation of Western blots from Fig 3.A (n=3). GAPDH and TUBB were used as loading and normalization controls. * P ≤0.05; ** P ≤0.01 compared with siNeg control. ND= undetected expression.
