# Supplementary material for: SOX9 Protein in Pancreatic Cancer Regulates Multiple Cellular Networks in a Cell-Specific Manner
Source: Biomedicines. 2022 Jun 21;10(7):1466. doi: 10.3390/biomedicines10071466 (PMC9312990; doi:10.3390/biomedicines10071466)
Supplement: Supplementary file 1 [file biomedicines-10-01466-s001.zip › biomedicines-1766456-supplementary proof/Figure S5.pdf]

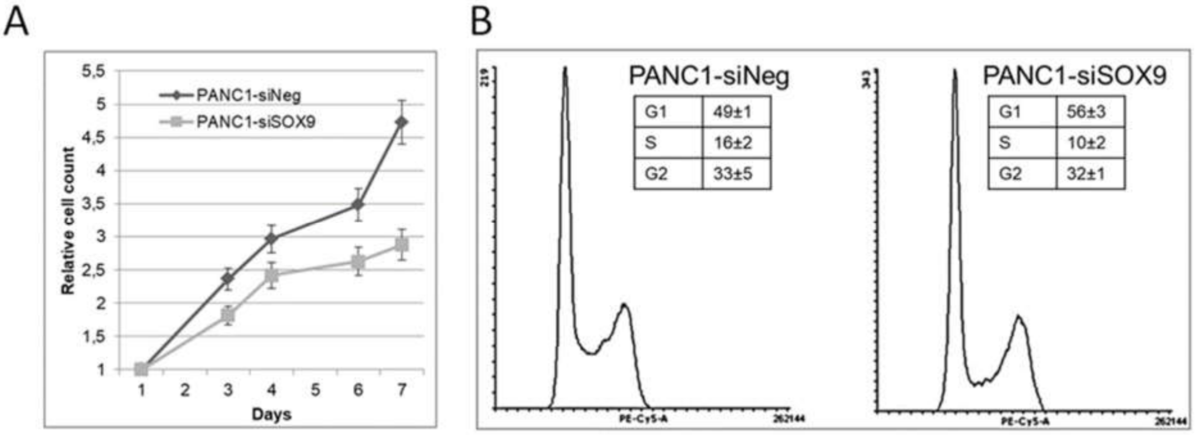

Figure S5. The effect of SOX9 downregulation on Panc1 cell proliferation. (A) Kinetics of cell growth during 7 days by MTS assay. Data are normalized relative to values on the first day of the experiment. (B) Flow cytometry results showed that the number of cells in the S stage of siSOX9 transfected Panc1 cells was lower than of siNeg transfected cells, whereas the number of cells in the G1 stage of siSOX9 transfected Panc1 cells was higher than that of siNeg transfected cells.
