# Supplementary material for: SOX9 Protein in Pancreatic Cancer Regulates Multiple Cellular Networks in a Cell-Specific Manner
Source: Biomedicines. 2022 Jun 21;10(7):1466. doi: 10.3390/biomedicines10071466 (PMC9312990; doi:10.3390/biomedicines10071466)
Supplement: Supplementary file 1 [file biomedicines-10-01466-s001.zip › biomedicines-1766456-supplementary proof/Figure S6.pdf]

A

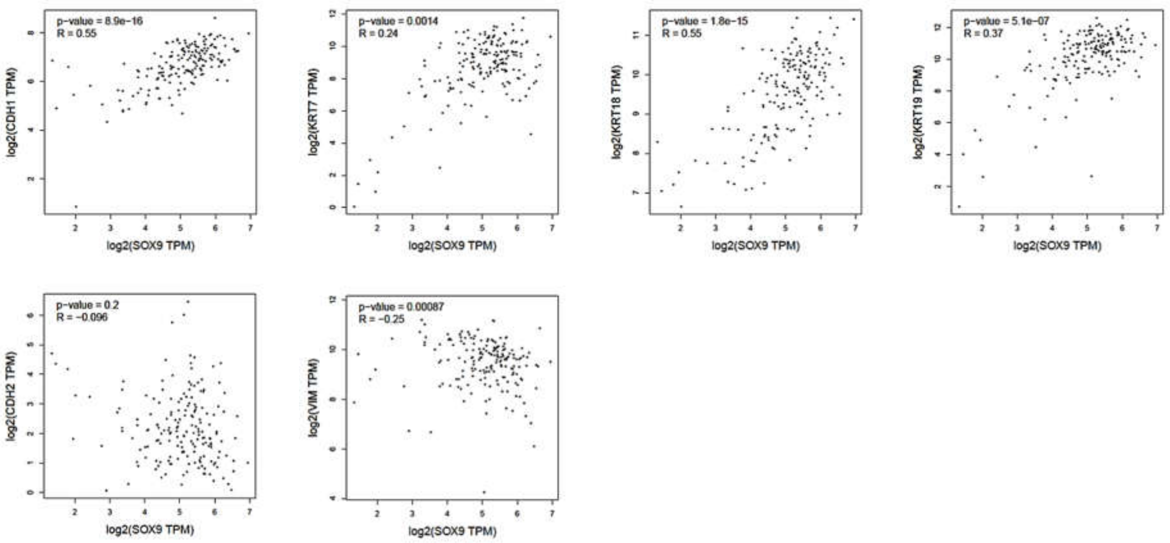

B

Cell lines	*SOX9*	*CDH1*	*KRT7*	*KRT18*	*KRT19*	*CDH2*	*VIM*
**SNU-324**	8	131	698	302	381	0	1
**T3M-4**	10	109	755	203	2417	0,7	1
**HPAC**	19	88	1210	927	2733	0	1
**Hs 766T**	26	5	708	741	2013	88	7
**BxPC-3**	31	359	2052	1064	3390	6	5
**DAN-G**	33	96	629	708	740	3	38
**HUP-T3**	34	205	523	253	1290	20	34
**SU. 86.86**	40	259	1067	1892	734	24	9
**Suit-2**	42	29	10	1038	1467	23	408
**HPAF-II**	43	284	2488	3082	4155	0	3
**SW 1990**	43	13	629	1011	467	12	604
**AsPC-1**	54	74	10	2306	2231	0	475
**PK-1**	56	264	2448	2450	2991	0	6
**Panc 10.05**	62	369	776	3997	3556	0	1
**PA-TU-8902**	66	165	605	1467	1162	2	3
**Panc 03.27**	66	132	2036	2309	2136	29	77
**HUP-T4**	67	484	1652	1670	4216	27	3
**TCC-PAN2**	73	52	16	729	2706	59	380
**PA-TU-8988S**	81	244	42	511	295	0	0,8
**Panc 04.03**	82	186	1775	1873	3785	0	2
**Panc-1**	84	34	5	1230	1943	75	1290
**MiaPaCa-2**	85	0,5	2	1143	613	0	2166
**Panc 05.04**	85	297	1552	2691	4300	21	7
**Capan-2**	86	478	1093	2688	2488	2	7
**PSN1**	98	8	792	547	656	0	391
**CFPAC-1**	100	205	557	1147	3301	66	303
**Capan-1**	103	164	797	1163	2437	10	1
**Panc 08.13**	114	329	1778	2046	4296	0	2
**SNU-213**	123	261	2186	677	3591	51	77
**R =**		**0,191**	**-0,023**	**0,287**	**0,256**	**0,075**	**0,246**
p=		0,26	0,642	0,288	0,108	0,404	0,284

Figure S6. Expression of SOX9 and EMT markers in pancreatic cancer tumors and cell lines. (A) Correlation analysis of SOX9 expression, epithelial phenotype and mesenchymal phenotype markers in pancreatic tumors of the TCGA cohort. (B) Correlation analysis of SOX9 expression, epithelial phenotype and mesenchymal phenotype markers in 29 pancreatic cancer cell lines. Gene expression values were obtained from the Expression Atlas database (www.ebi.ac.uk/gxa/home) and presented as TPM (transcripts per million). Correlation coefficient (R) have been calculated using the Pearson method.
