# Supplementary material for: SOX9 Protein in Pancreatic Cancer Regulates Multiple Cellular Networks in a Cell-Specific Manner
Source: Biomedicines. 2022 Jun 21;10(7):1466. doi: 10.3390/biomedicines10071466 (PMC9312990; doi:10.3390/biomedicines10071466)
Supplement: Supplementary file 1 [file biomedicines-10-01466-s001.zip › biomedicines-1766456-supplementary proof/Supplementary Figure Legends.pdf]

**Supplementary Figure Legends**

**Figure S1.** Immunofluorescence imaging of SOX9 expression in AsPC-1, BxPC-3, Colo357, Capan-2, MiaPaCa-2 and Panc1 cells. Cells were stained for total cytokeratin (green) and SOX9 (red). Nuclei were stained with DAPI (blue). Scale bar = 100 μm.

**Figure S2.** (A) Western blot analysis of SOX9 expression in Panc1 cells transfected with control siNeg, three different siRNAs and their equimolar mix (siSOX9). GAPDH was used as loading and normalization control. (B) Densitometric quantitation of Western blots from SF2A. (C) Morphological changes in Panc1 cancer cells induced by siSOX9 transfection. Phase contrast. Scale bar = 100 μm

**Figure S3.** Densitometric quantitation of Western blots from Fig 2.A, C, E and G (n=3). GAPDH and TUBB were used as loading and normalization controls. * $P \leq 0.05$; ** $P \leq 0.01$ compared with siNeg control. ND= undetected expression.

**Figure S4.** Densitometric quantitation of Western blots from Fig 3.A (n=3). GAPDH and TUBB were used as loading and normalization controls. * $P \leq 0.05$; ** $P \leq 0.01$ compared with siNeg control. ND= undetected expression.

**Figure S5.** The effect of SOX9 downregulation on Panc1 cell proliferation. A. Kinetics of cell growth during 7 days by MTS assay. Data are normalized relative to values on the first day of the experiment. B. Flow cytometry results showed that the number of cells in the S stage of siSOX9 transfected Panc1 cells was lower than of siNeg transfected cells, whereas the number of cells in the G1 stage of siSOX9 transfected Panc1 cells was higher than that of siNeg transfected cells.

**Figure S6.** Expression of SOX9 and EMT markers in pancreatic cancer tumors and cell lines. (A) Correlation analysis of SOX9 expression, epithelial phenotype and mesenchymal phenotype markers in pancreatic tumors of the TCGA cohort. (B) Correlation analysis of SOX9 expression, epithelial phenotype and mesenchymal phenotype markers in 29 pancreatic cancer cell lines. Gene expression values were obtained from the Expression Atlas database

(www.ebi.ac.uk/gxa/home) and presented as TPM (Transcripts Per Million). Correlation coefficient (R) have been calculated using the Pearson method.
