# Supplementary material for: SOX9 Protein in Pancreatic Cancer Regulates Multiple Cellular Networks in a Cell-Specific Manner
Source: Biomedicines. 2022 Jun 21;10(7):1466. doi: 10.3390/biomedicines10071466 (PMC9312990; doi:10.3390/biomedicines10071466)
Supplement: Supplementary file 1 [file biomedicines-10-01466-s001.zip › biomedicines-1766456-supplementary proof/Table S1.pdf]

Table S1. List of the antibodies used for Western Blots and immunofluorescence imaging.

Primary antibodies:

	Name	Company	Host	Dilution
1	Anti-SOX9 (D8G8H)	Cell Signaling Technology, USA	Rabbit	WB 1:1000<br>IF 1:200
2	Anti-GAPDH (0411)	Santa Cruz Biotechnology, USA	Mouse	WB 1:2500
3	Anti-β-Tubulin (9F3)	Cell Signaling Technology, USA	Rabbit	WB 1:1000
4	Anti-E-Cadherin (H-108)	Santa Cruz Biotechnology, USA	Rabbit	WB 1:500
5	Anti-P-Cadherin (A-10)	Santa Cruz Biotechnology, USA	Mouse	WB 1:500
6	Anti-CK7 (C9097)	US Biological, USA	Mouse	WB 1:2000
7	Anti-CK18 (DC-10)	Santa Cruz Biotechnology, USA	Mouse	WB 1:1000
8	Anti-CK19 (BA17)	Cell Signaling Technology, USA	Mouse	WB 1:2000
9	Anti-Pan-cytokeratin (C11) FITC- labeled	Sigma-Aldrich, USA	Mouse	IF 1:200
10	Anti-α-E-Catenin (D9R5E)	Cell Signaling Technology, USA	Rabbit	WB 1:1000
11	Anti-β-Catenin (H-102)	Santa Cruz Biotechnology, USA	Rabbit	WB 1:500
12	Anti-Non-phospho (Active) β-Catenin (Ser45)	Cell Signaling Technology, USA	Rabbit	WB 1:1000
13	Anti-Catenin δ-1 (D7S2M)	Cell Signaling Technology, USA	Rabbit	WB 1:1000
14	Anti-γ-Catenin (D9M1Q)	Cell Signaling Technology, USA	Rabbit	WB 1:1000
15	Anti-N-Cadherin (D4R1H)	Cell Signaling Technology, USA	Rabbit	WB 1:1000
16	Anti-Vimentin (V9)	Sigma-Aldrich, USA	Mouse	WB 1:2500
17	Anti-Snail (L70G2)	Cell Signaling Technology, USA	Mouse	WB 1:1000
18	Anti-Slug (C40C6)	Cell Signaling Technology, USA	Mouse	WB 1:1000
19	Anti-ZEB1 (D80D3)	Cell Signaling Technology, USA	Rabbit	WB 1:1000
20	Anti-HNF1α (SAB2702189)	Sigma-Aldrich, USA	Mouse	WB 1:1000
21	Anti-GATA-4 (D3A3M)	Cell Signaling Technology, USA	Rabbit	WB 1:1000
22	Anti-GATA-6 (D61E4)	Cell Signaling Technology, USA	Rabbit	WB 1:1000
23	Anti-FoxA1/HNF3α (D7P9B)	Cell Signaling Technology, USA	Rabbit	WB 1:1000
24	Anti-FoxA2/HNF3β (D56D6)	Cell Signaling Technology, USA	Rabbit	WB 1:1000
25	Anti-Pdx1 (D59H3)	Cell Signaling Technology, USA	Rabbit	WB 1:1000
26	Anti-PTF1A (C-7)	Santa Cruz Biotechnology, USA	Mouse	WB 1:250
27	Anti-PCNA (PC10)	Cell Signaling Technology, USA	Mouse	WB 1:1000
28	Anti-Cyclin D1 (DCS-6)	Santa Cruz Biotechnology, USA	Mouse	WB 1:500
29	Anti-Cyclin D3 (DCS22)	Cell Signaling Technology, USA	Rabbit	WB 1:1000
30	Anti-Cyclin B1 (D5C10)	Cell Signaling Technology, USA	Rabbit	WB 1:1000
31	Anti-Survivin (FL-142)	Santa Cruz Biotechnology, USA	Rabbit	WB 1:500
32	Anti-p21 Waf1/Cip1 (CP74)	Sigma-Aldrich, USA	Mouse	WB 1:2000<br>IF 1:100
33	Anti-p27 Kip1 (D69C12)	Cell Signaling Technology, USA	Rabbit	WB 1:1000
34	Anti-BMI1 (D20B7)	Cell Signaling Technology, USA	Rabbit	WB 1:1000
35	Anti-P53 (DO-1)	Santa Cruz Biotechnology, USA	Mouse	WB 1:1000
36	Anti-PTEN (D4.3)	Cell Signaling Technology, USA	Rabbit	WB 1:1000

Secondary antibodies:

	Name	Company	Host	Dilution
1	Anti-rabbit IgG, HRP-linked	Cell Signaling Technology, USA	Goat	WB 1:1000
2	Anti-mouse IgG, HRP-linked	Cell Signaling Technology, USA	Horse	WB 1:1000
3	Anti-rabbit IgG, Alexa Fluor 555	Invitrogen, USA	Goat	IF 1:100
4	Anti-mouse IgG, Alexa Fluor 555	Invitrogen, USA	Goat	IF 1:100
