# Supplementary material for: SOX9 Protein in Pancreatic Cancer Regulates Multiple Cellular Networks in a Cell-Specific Manner
Source: Biomedicines. 2022 Jun 21;10(7):1466. doi: 10.3390/biomedicines10071466 (PMC9312990; doi:10.3390/biomedicines10071466)
Supplement: Supplementary file 1 [file biomedicines-10-01466-s001.zip › biomedicines-1766456-supplementary proof/Table S2.pdf]

Table S2. List of the siRNAs used for targeting human *SOX9* RNA.

siRNA	Target	Sense Sequence (5′ to 3′)
**si#18**	**5′UTR**	**5′-GCUCGAAACTGACUGGAAAdTdT-3′**
**si#1299**	**ORF**	**5′-UCGAGACCUUCGAUGUCAAdTdT-3′**
**si#2691**	**3′UTR**	**5′-GAAGCAUUUGGUAAGCUUUdTdT-3′**
