# Supplementary material for: SOX9 Protein in Pancreatic Cancer Regulates Multiple Cellular Networks in a Cell-Specific Manner
Source: Biomedicines. 2022 Jun 21;10(7):1466. doi: 10.3390/biomedicines10071466 (PMC9312990; doi:10.3390/biomedicines10071466)
Supplement: Supplementary file 1 [file biomedicines-10-01466-s001.zip › biomedicines-1766456-supplementary proof/Table S3.pdf]

Table S3. List of the primers used for RT-qPCR.

Gene	Primer sequence (5′ to 3′)	PCR product
SOX9	**FW: 5′- AGCTCTGGAGACTTCTGAACGA -3′**<br>**RW: 5′- TAGCTGCCCGTGTAGGTGAC -3′**	**535 bp**
SNAI1	**FW: 5′- CCAATCGGAAGCCTAACTAC -3′**<br>**RW: 5′- GCGGTGGGGTTGAGGATCTC -3′**	**124 bp**
SNAI2	**FW: 5′- AGAAGGTTTTGGAGCAGTTTTTG -3′**<br>**RW: 5′- TGGTTGCTTCAAGGACACAT -3′**	**160 bp**
FOXA2	**FW: 5′- GAGACTTTGGGGAGACGGTG -3′**<br>**RW: 5′- GTTTGGGACGGAACGGCTGC -3′**	**118 bp**
GATA4	**FW: 5′- GTGTCAACTGTGGGGCTATG -3′**<br>**RW: 5′- GGTGGTGGTGGTCTGGCAG -3′**	**188 bp**
CDKN1A	**FW: 5′- CGGATTAGGGCTTCCTCTTGG -3′**<br>**RW: 5′- GACCATGTGGACCTGTCACTG -3′**	**175 bp**
TP53	**FW: 5′- CTCCTCAGCATCTTATCCGAGT -3′**<br>**RW: 5′- AGAGGAGCTGGTGTTGTTGG -3′**	**377 bp**
PTEN	**FW: 5′- TGGAGTTTACCGGCAGCATC -3′**<br>**RW: 5′- TAGTGCACAGTTCCACCCCT -3′**	**192 bp**
HPRT	**FW: 5′- GCTATAAATTCTTTGCTGACCTGCTG -3′**<br>**RW: 5′- AATTACTTTTATGTCCCCTGTTGACTGG -3′**	**140 bp**
