# Supplementary material for: SOX9 Protein in Pancreatic Cancer Regulates Multiple Cellular Networks in a Cell-Specific Manner
Source: Biomedicines. 2022 Jun 21;10(7):1466. doi: 10.3390/biomedicines10071466 (PMC9312990; doi:10.3390/biomedicines10071466)
Supplement: Supplementary file 1 [file biomedicines-10-01466-s001.zip › biomedicines-1766456-supplementary proof/Table S4.pdf]

Table S4. Results of two independent experiments demonstrated that the downregulation of SOX9 decreased the migratory activity of Panc1-EGFP cells transplanted into *Danio rerio* embryos.

	**Panc1-EGFP/siNeg**	**Panc1-EGFP/siSOX9**
1 Exp	11(2)	11(0)
2 Exp	80(7)	78(1)
**Total**	**91 (9)**	**89(1)**
