# Supplementary material for: SOX9 Protein in Pancreatic Cancer Regulates Multiple Cellular Networks in a Cell-Specific Manner
Source: Biomedicines. 2022 Jun 21;10(7):1466. doi: 10.3390/biomedicines10071466 (PMC9312990; doi:10.3390/biomedicines10071466)
Supplement: Supplementary file 1 [file biomedicines-10-01466-s001.zip › biomedicines-1766456-supplementary proof/Figure S2.pdf]

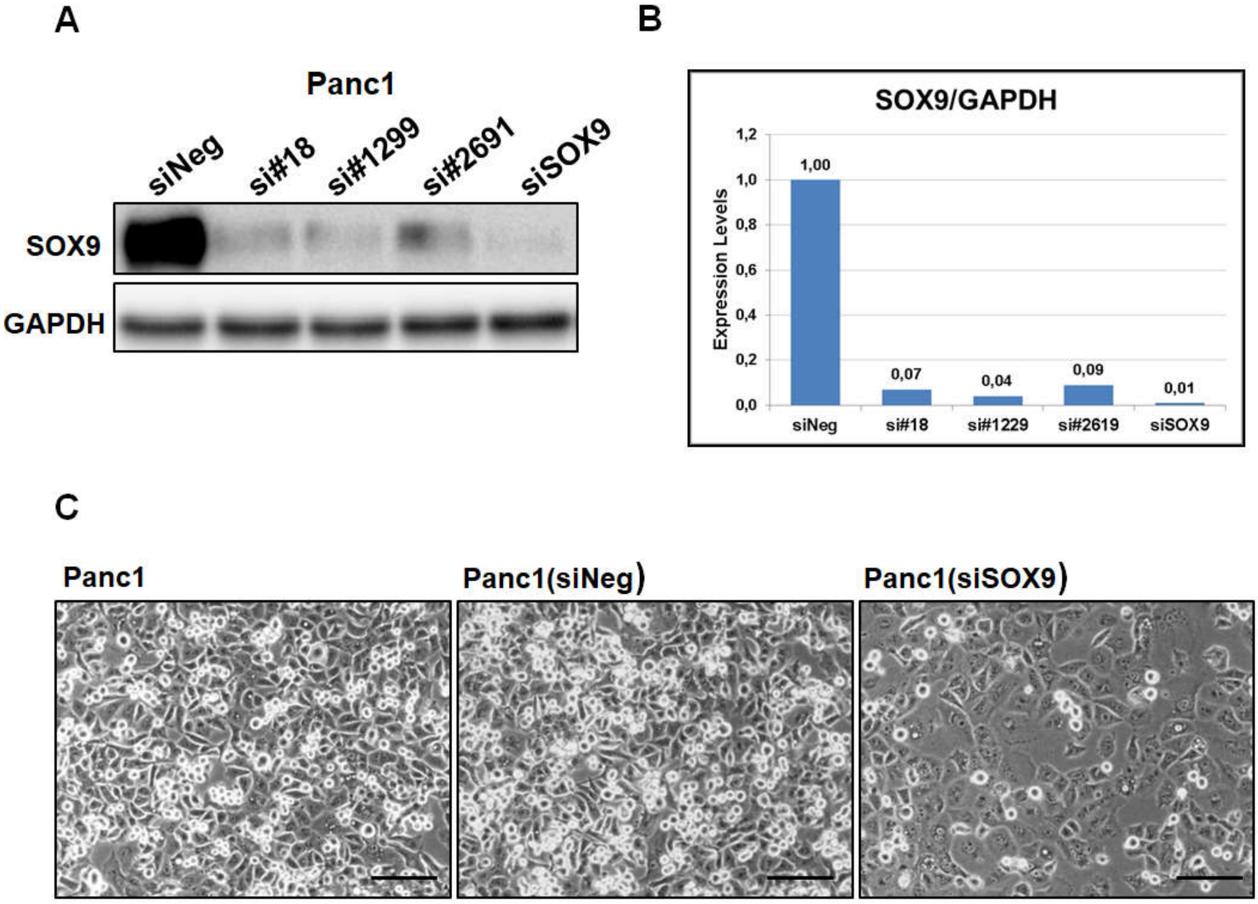

Figure S2. (A) Western blot analysis of SOX9 expression in Panc1 cells transfected with control siNeg, three different siRNAs and their equimolar mix (siSOX9). GAPDH was used as loading and normalization control. (B) Densitometric quantitation of Western blots from SF2A. (C) Morphological changes in Panc1 cancer cells induced by siSOX9 transfection. Phase contrast. Scale bar = 100 µm
