# Supplementary material for: SOX9 Protein in Pancreatic Cancer Regulates Multiple Cellular Networks in a Cell-Specific Manner
Source: Biomedicines. 2022 Jun 21;10(7):1466. doi: 10.3390/biomedicines10071466 (PMC9312990; doi:10.3390/biomedicines10071466)
Supplement: Supplementary file 1 [file biomedicines-10-01466-s001.zip › biomedicines-1766456-supplementary proof/Figure S3.pdf]

A

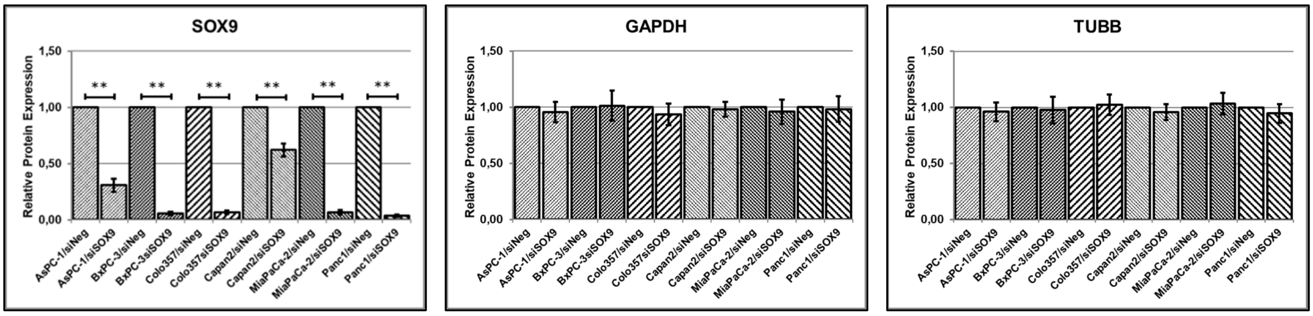
SOX9
GAPDH
TUBB
Relative Protein Expression
1,50
1,00
0,50
0,00
**
AsPC-1/siNeg
AsPC-1/siSOX9
BxPC-3/siNeg
BxPC-3/siSOX9
Colo357/siNeg
Colo357/siSOX9
Capan2/siNeg
Capan2/siSOX9
MiaPaCa-2/siNeg
MiaPaCa-2/siSOX9
Panc1/siNeg
Panc1/siSOX9

C

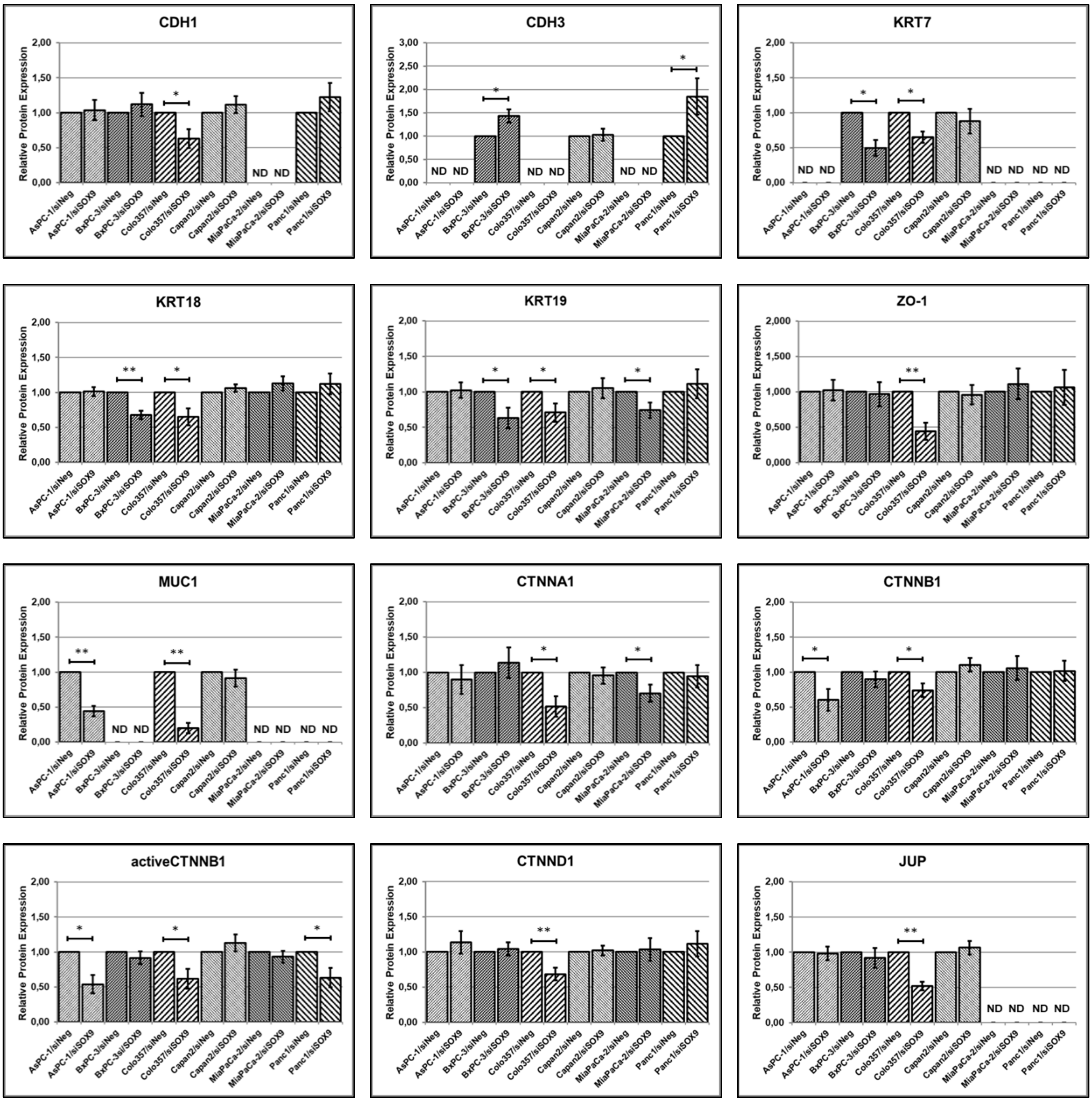
CDH1
CDH3
KRT7
KRT18
KRT19
ZO-1
MUC1
CTNNA1
CTNNB1
activeCTNNB1
CTNND1
JUP
Relative Protein Expression
ND
*
**
AsPC-1/siNeg
AsPC-1/siSOX9
BxPC-3/siNeg
BxPC-3/siSOX9
Colo357/siNeg
Colo357/siSOX9
Capan2/siNeg
Capan2/siSOX9
MiaPaCa-2/siNeg
MiaPaCa-2/siSOX9
Panc1/siNeg
Panc1/siSOX9

E

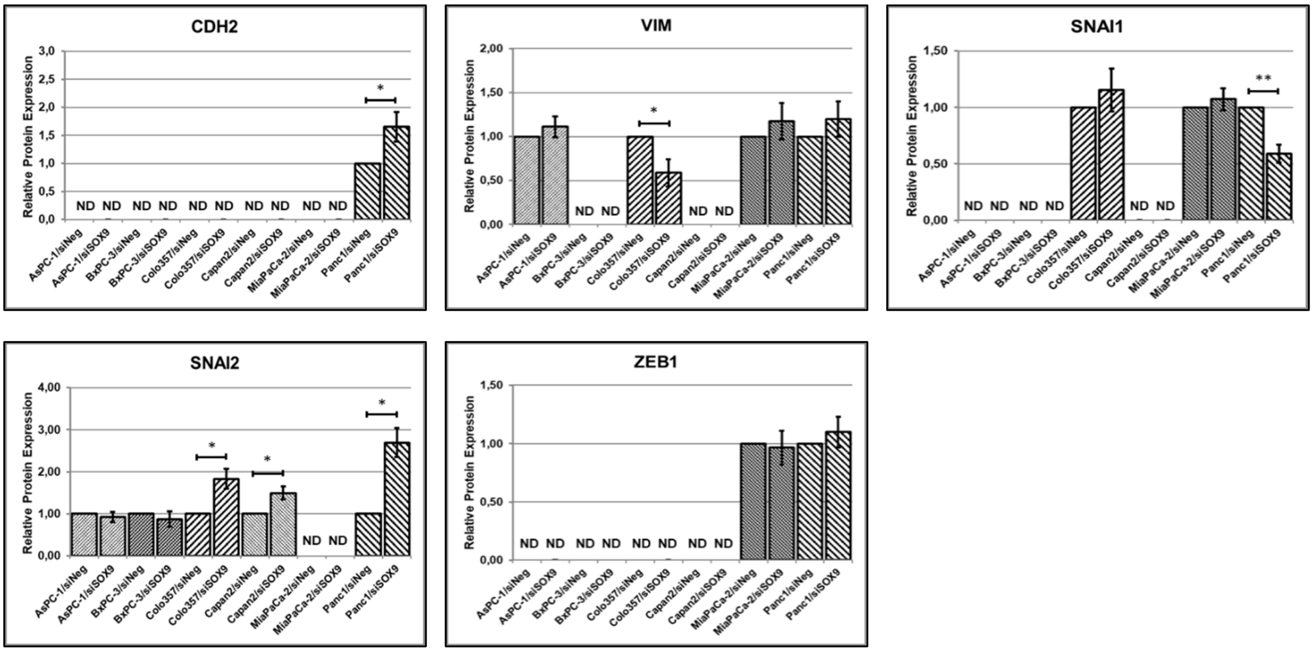

G

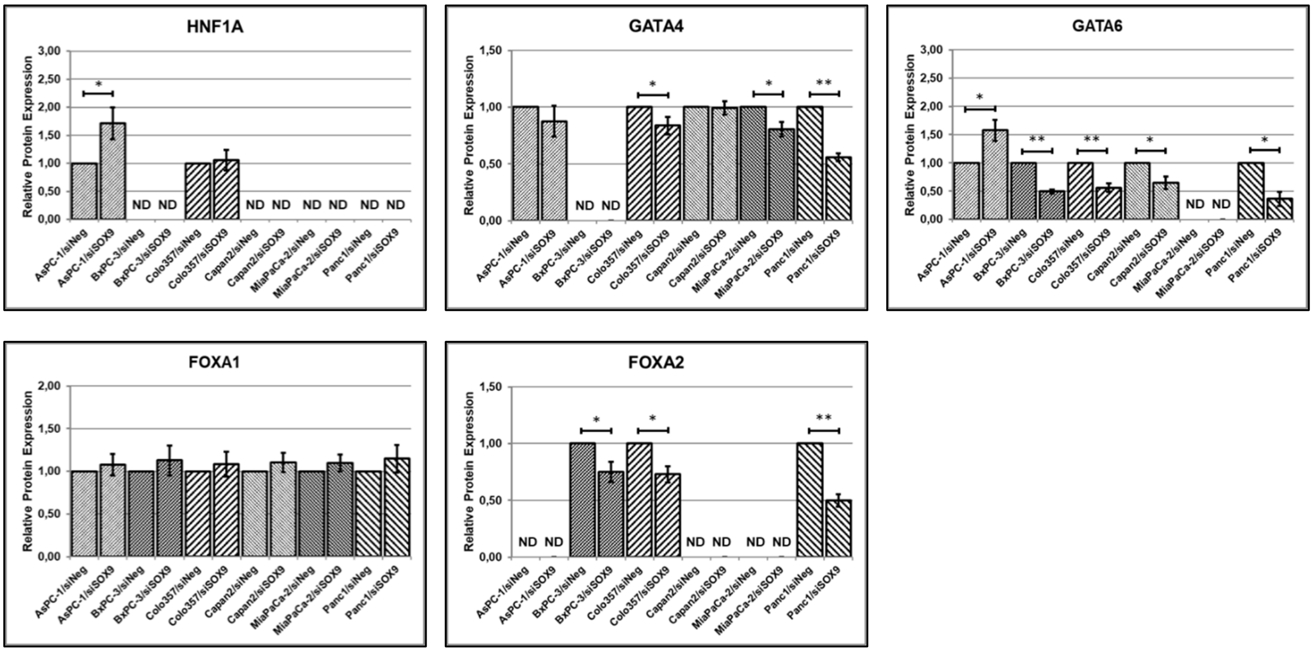

Figure S3. Densitometric quantitation of Western blots from Fig 2.A, C, E and G (n=3). GAPDH and TUBB were used as loading and normalization controls. * P ≤0.05; ** P ≤0.01 compared with siNeg control. ND= undetected expression.
