# Supplementary material for: SOX9 Protein in Pancreatic Cancer Regulates Multiple Cellular Networks in a Cell-Specific Manner
Source: Biomedicines. 2022 Jun 21;10(7):1466. doi: 10.3390/biomedicines10071466 (PMC9312990; doi:10.3390/biomedicines10071466)
Supplement: Supplementary file 1 [file biomedicines-10-01466-s001.zip › biomedicines-1766456-supplementary proof/Figure S1.pdf]

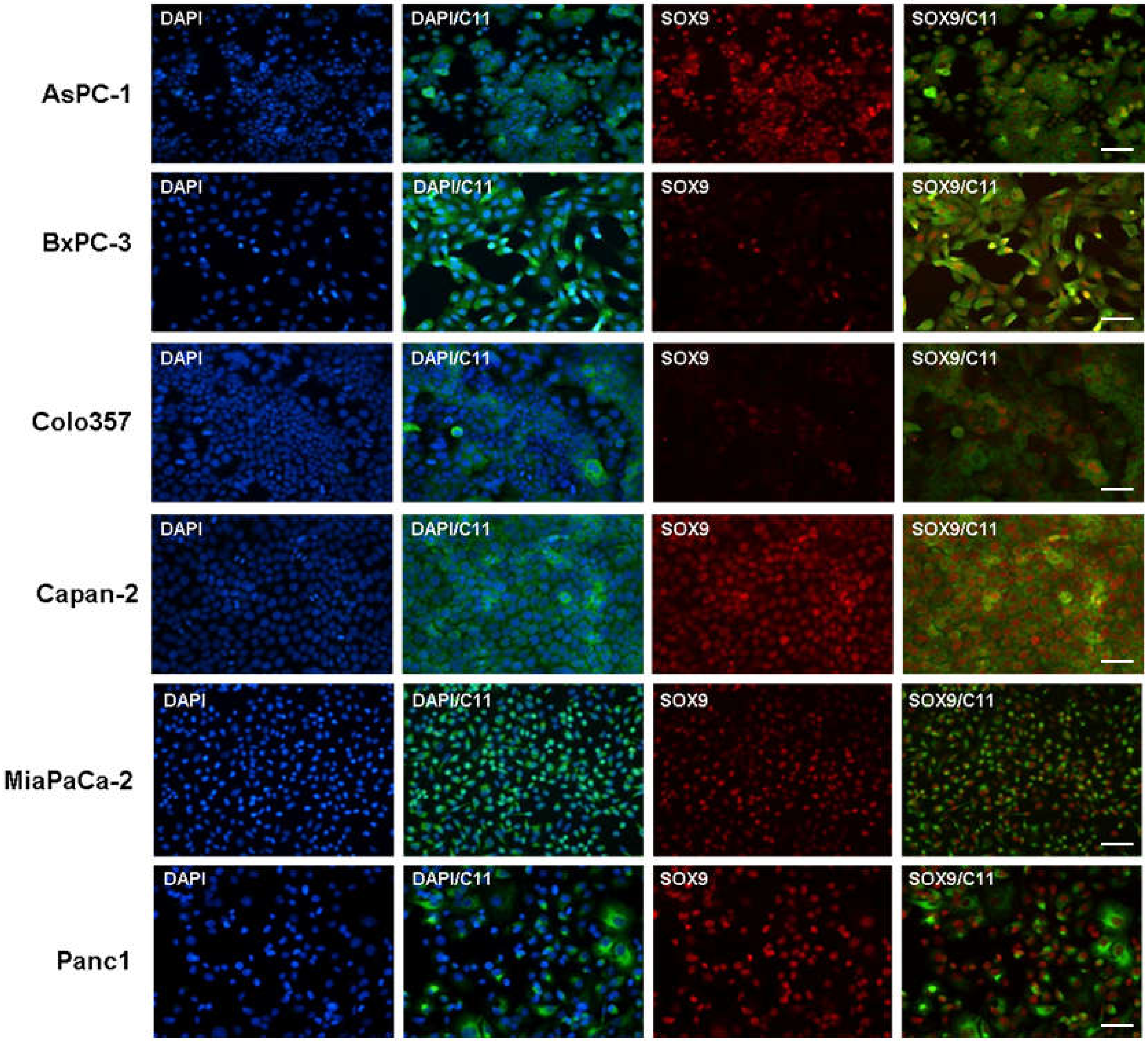

Figure S1. Immunofluorescence imaging of SOX9 expression in AsPC-1, BxPC-3, Colo357, Capan-2, MiaPaCa-2 and Panc1 cells. Cells were stained for total cytokeratin (green) and SOX9 (red). Nuclei were stained with DAPI (blue). Scale bar = 100 μm.
